# Feeding height stratification among the herbivorous dinosaurs from the Dinosaur Park Formation (upper Campanian) of Alberta, Canada

**DOI:** 10.1186/1472-6785-13-14

**Published:** 2013-04-04

**Authors:** Jordan C Mallon, David C Evans, Michael J Ryan, Jason S Anderson

**Affiliations:** 1 Department of Biological Sciences, University of Calgary, Calgary, Alberta, T2N 1N4, Canada; 2 Department of Natural History, Royal Ontario Museum, 100 Queen's Park, Toronto, ON, M5S 2C6, Canada; 3 Department of Vertebrate Paleontology, Cleveland Museum of Natural History, 1 Wade Oval Drive, University Circle, Cleveland, Ohio, 44106, USA; 4 Department of Comparative Biology & Experimental Medicine, University of Calgary, Calgary, Alberta, T2N 4N1, Canada; 5 Current address: Palaeobiology, Canadian Museum of Nature, STN “D”, P. O. Box 3443, Ottawa, ON, Canada

**Keywords:** Dinosaur Park Formation, Feeding height stratification, Niche partitioning, Evolutionary palaeoecology, Ankylosauria, Ceratopsidae, Hadrosauridae

## Abstract

**Background:**

Herbivore coexistence on the Late Cretaceous island continent of Laramidia has been a topic of great interest, stemming from the paradoxically high diversity and biomass of these animals in relation to the relatively small landmass available to them. Various hypotheses have been advanced to account for these facts, of which niche partitioning is among the most frequently invoked. However, despite its wide acceptance, this hypothesis has not been rigorously tested. This study uses the fossil assemblage from the Dinosaur Park Formation of Alberta as a model to investigate whether niche partitioning facilitated herbivorous dinosaur coexistence on Laramidia. Specifically, the question of feeding height stratification is examined in light of the role it plays in facilitating modern ungulate coexistence.

**Results:**

Most herbivorous dinosaur species from the Dinosaur Park Formation were restricted to feeding no higher than approximately 1 m above the ground. There is minimal evidence for feeding height partitioning at this level, with ceratopsids capable of feeding slightly higher than ankylosaurs, but the ecological significance of this is ambiguous. Hadrosaurids were uniquely capable of feeding up to 2 m quadrupedally, or up to 5 m bipedally. There is no evidence for either feeding height stratification within any of these clades, or for change in these ecological relationships through the approximately 1.5 Ma record of the Dinosaur Park Formation.

**Conclusions:**

Although we cannot reject the possibility, we find no good evidence that feeding height stratification, as revealed by reconstructed maximum feeding heights, played an important role in facilitating niche partitioning among the herbivorous dinosaurs of Laramidia. Most browsing pressure was concentrated in the herb layer, although hadrosaurids were capable of reaching shrubs and low-growing trees that were out of reach from ceratopsids, ankylosaurs, and other small herbivores, effectively dividing the herbivores in terms of relative abundance. Sympatric hadrosaurids may have avoided competing with one another by feeding differentially using bipedal and quadrupedal postures. These ecological relationships evidently proved to be evolutionarily stable because they characterize the herbivore assemblage of the Dinosaur Park Formation through time. If niche partitioning served to facilitate the rich diversity of these animals, it may have been achieved by other means in addition to feeding height stratification. Consideration of other feeding height proxies, including dental microwear and skull morphology, may help to alleviate problems of underdetermination identified here.

## Background

During the Late Cretaceous, a shallow inland sea divided North America into two longitudinally-arrayed landmasses. The eastern landmass—called Appalachia [1]—supported an enigmatic fauna known only from scant fossil remains [2]. The western landmass—called Laramidia [1]—supported a rich diversity of herbivorous dinosaurs. Included among these were such small forms (< 100 kg) as hypsilophodontids, pachycephalosaurids, and leptoceratopsids. Putatively facultative herbivores (or omnivores) included troodontids, oviraptorosaurs, ornithomimids, and therizinosaurids [3]. The megaherbivorous dinosaurs—primarily represented by ankylosaurs, ceratopsids, and hadrosaurids—form the most significant component of this fauna [4-6], in terms of both population (75–82% of total dinosaur fauna; [7]) and body size.

This high diversity of contemporaneous herbivores in Laramidia has long puzzled investigators. Laramidia had a total estimated area between just 4 million km^2^[8] and 7.7 million km^2^[5], and dinosaur distribution was segregated into distinct northern and southern faunal provinces that presumably reflect strict habitat preferences linked to a palaeoclimatic gradient [4-6,9]. By contrast, the area of sub-Saharan Africa, where living vertebrate herbivores are most diverse, is approximately 23.6 million km^2^, with the highest diversity occurring in the East African savannah [10]. The diverse herbivorous dinosaur fauna of Laramidia therefore appears to have been spatially restricted compared to modern analogs, and this likely would have increased the potential for resource competition. The problem of the coexistence of these dinosaurs is further exaggerated when considering the presumably large nutritional requirements of the megaherbivorous forms [11-14], as well as their high population densities [15-22], both of which would have placed increased pressure on the resource base.

Two main hypotheses have been proposed to account for the coexistence of so many herbivores in Laramidia. The first hypothesis contends that plant resources were simply not limiting, a scenario that could be explained by numerous causal factors. For example, it may have been that, in spite of the fact that the nutritional requirements of the megaherbivorous dinosaurs were absolutely high, the inferred bradymetabolic thermoregulatory systems of these animals imparted relatively low nutritional requirements compared to their mammalian counterparts, minimizing pressure on the resource base [5,14,23]. Alternatively, plant resources may not have been limiting because primary productivity was elevated during the Late Cretaceous [23,24]. This suggestion was also advanced to account for the increased abundance of browsers during the Miocene [25,26]. Increased primary productivity may have resulted in part from the prevalence of marginal coastal environments during the Late Cretaceous [27]; however, palaeoclimatological modeling [28,29] and experimental evidence [30] also suggest that elevated atmospheric pressure and CO_2_ during the Late Cretaceous would have yielded similar effects. A third option might be that predation pressure from tyrannosaurids and dromaeosaurids was sufficiently high during the Late Cretaceous to suppress herbivore population densities, leading to reduced pressure on the resource base as in the first scenario. A similar mechanism is thought to help shape the structure of modern African ungulate communities [31].

The second hypothesis maintains that plant resources were limiting, and that herbivorous dinosaur coexistence was facilitated by dietary niche partitioning [6,13,32]. If so, these animals should have been adapted in such a way as to avoid competing with one another, perhaps varying in morphology so as to specialize on different plant types [7,8,33-42]. Numerous ecomorphological correlates among the herbivores have been hypothesized to reflect niche partitioning, including differences in snout shape [7,33,34,36-41,43,44], jaw mechanics and tooth shape [36,40,45,46], and feeding height stratification [12,13,33,34].

The late Campanian-aged Dinosaur Park Formation (DPF) of Alberta preserves the most diverse dinosaur assemblage currently known, including the most diverse assemblage of large-bodied herbivores from Laramidia [47]. Currently, approximately 33 herbivorous and omnivorous dinosaur species are recognized [48]. Biostratigraphic work shows that these taxa are distributed heterogeneously throughout the formation, such that different species are restricted to different horizons within the formation [47,49-51]. Although not all 33 taxa co-existed, over half likely co-existed in certain time intervals, including at least eight megaherbivorous taxa (Megaherbivore Assemblage Zone 1 [51]). Therefore, this assemblage provides a unique opportunity to evaluate possible dietary niche partitioning among Late Cretaceous herbivorous dinosaurs.

Niche partitioning via feeding height stratification is thought to have facilitated sauropod diversity during the Late Jurassic [33,34,44,52-58]. Likewise, some [12,13,33] have suggested that the rich diversity of herbivorous dinosaurs in the Late Cretaceous of Western Interior North America was fostered, in part, by a similar mechanism, but rigorous quantification of these patterns and formal tests of specific hypotheses are lacking. The aim of this paper is to test the hypothesis that the long-term coexistence of these animals was facilitated by dietary niche partitioning, more specifically via feeding height stratification.

## Methods

We examined the question of feeding height stratification by estimating the maximum feeding heights (MFHs) of the dinosaurs from the DPF. It is prudent to note from the outset that MFH does not necessarily reflect habitual feeding height. Habitual feeding height is very difficult to determine in fossil forms, particularly in light of the fact that amniotes rarely maintain the cervical column in an osteologically neutral pose [59]. Regardless, most discussions to date regarding the feeding habits of dinosaurs from the DPF have focused on MFH, which is our primary concern. See Discussion below for further treatment of maximal and habitual feeding heights.

The data used in this study are listed in Additional file 1. We excluded juvenile specimens, identified by their small size and/or under-developed cranial ornamentation, because we were specifically interested in estimating the MFHs of fully-grown individuals. Presumably, juveniles fed at intermediate heights and their inclusion in this study would have no effect on the calculation of MFHs. We also excluded the facultatively herbivorous/omnivorous theropods because they formed only a small component of the DPF fauna [7,12,60,61], and were therefore unlikely to have consumed an appreciable amount of plant matter. Many of these animals are also known only from fragmentary remains, and their MFHs can be estimated only with a great deal of uncertainty.

We studied all fossil specimens with permission from the following institutions: ACM, Beneski Museum of Natural History, Amherst College, Amherst; AMNH, American Museum of Natural History, New York; CMN, Canadian Museum of Nature, Ottawa; MCSNM, Museo Civico di Storia Naturale di Milano, Milan; NSM PV, National Museum of Nature and Science, Tokyo; ROM, Royal Ontario Museum, Toronto; TMP, Royal Tyrrell Museum of Palaeontology, Drumheller; UALVP, University of Alberta Laboratory of Vertebrate Palaeontology, Edmonton; YPM, Yale Peabody Museum, New Haven.

### Maximum feeding height estimation

**Figure 1:**
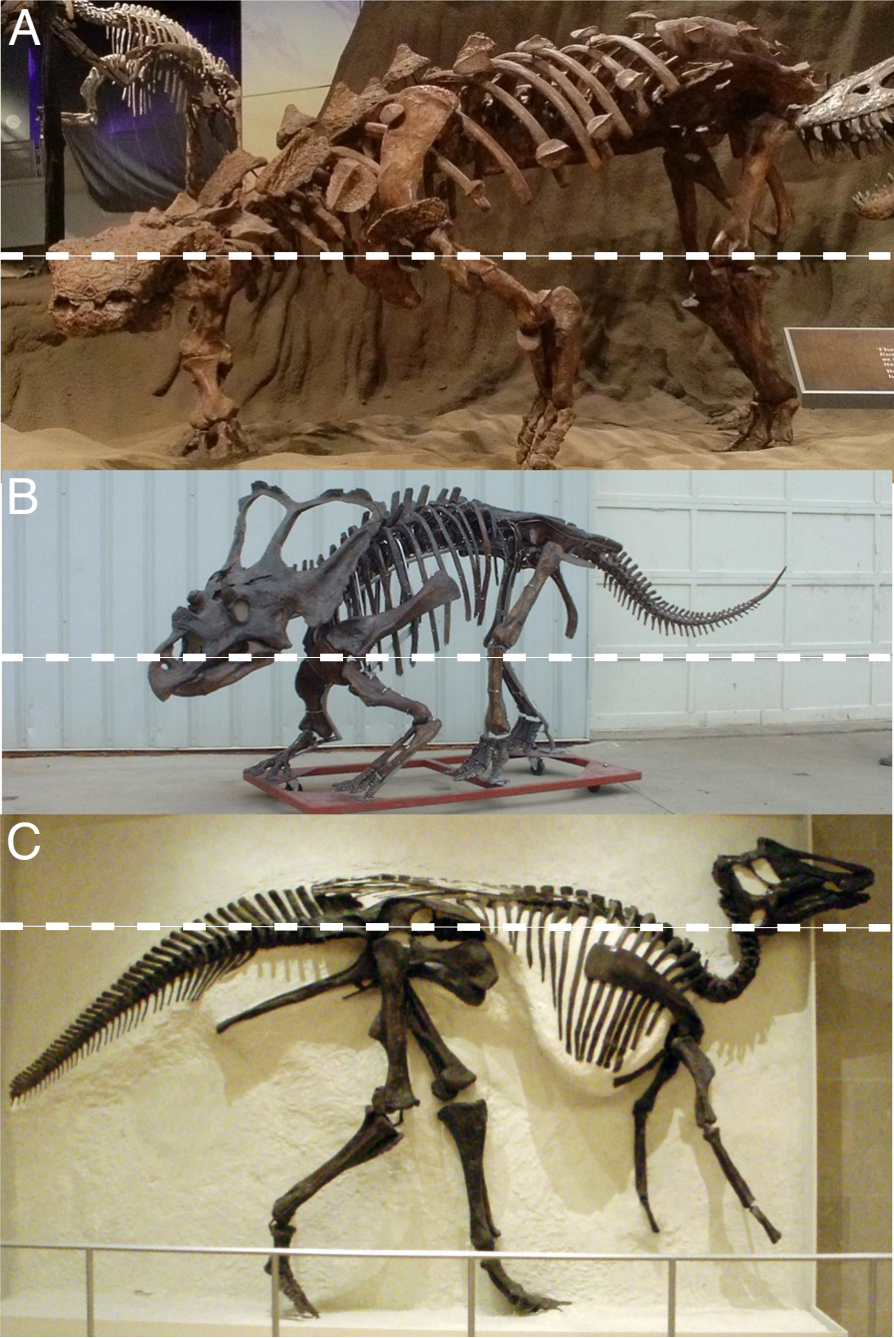
**Quadrupedal MFH proxies used in this study.** Shoulder height (calculated as the combined lengths of the humerus, radius, and metacarpal III) is used to approximate the MFHs of ankylosaurs (**A**), ceratopsids (**B**), and other small, quadrupedal forms. Hip height (calculated as the combined lengths of the femur, tibia/fibula, and metatarsal III) is used to approximate the quadrupedal MFHs of hadrosaurids (**C**). Dashed lines indicate the approximate quadrupedal MFH. Image (**B**) provided by G. Danis (used with permission).

When estimating the MFHs of the herbivorous dinosaurs, it was important to devise some metric that allowed for the use of both articulated and disarticulated skeletons, thereby maximizing sample size. MFH can often be measured directly from mounted specimens, but many reasonably complete skeletons remain disarticulated in museum collections. For this reason, we calculated MFH as follows: for the quadrupedal forms, we estimated MFH from shoulder height, calculated by adding the lengths of the humerus, radius, and metacarpal III. Observations of mounted skeletons reveal that this is an appropriate proxy for MFH because the mouth is approximately level with the glenoid of the shoulder (Figure 1A, B). In the case of ceratopsids, the mouth could not be brought much higher than this because the extension of the head at the atlanto-occipital joint would eventually cause the huge parietosquamosal frill to abut the dorsal surface of the shoulders (Figure 1B). For hadrosaurids, quadrupedal MFH was estimated from hip height, calculated by summing the lengths of the femur, tibia, and metatarsal III (the fibula was used if the tibia was not available). Again, observations of mounted hadrosaurid skeletons confirm that this is an appropriate proxy for quadrupedal MFH (Figure 1C).

**Figure 2:**
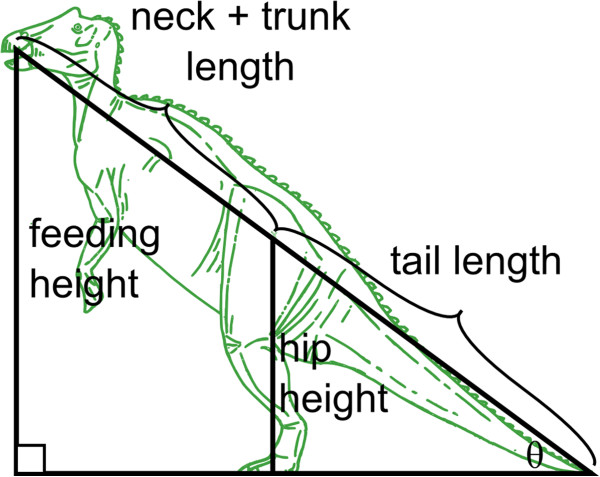
**Depiction of the trigonometric model used here to estimate bipedal MFHs of hadrosaurids.** See Methods for details.

For bipedal forms (including the facultatively bipedal hadrosaurids and leptoceratopsid), bipedal MFH was calculated using trigonometry: assuming that the animal in question could rear up on its hind legs only so far as the distal tip of its tail would touch the ground (a reasonable assumption, given that the tails of many of these animals were relatively inflexible due the presence of either tendon trellises or ossified myorhabdoi [62] that extended much of their lengths), a right triangle is formed (Figure 2) with the vertical side equal to hip height and the hypotenuse equal to tail length. The intersection of the hypotenuse and the horizontal side of the triangle forms angle θ, which can be calculated as θ = sin^-1^(hip height/tail length). The triangle can then be extended by adding the combined length of the trunk and neck to the hypotenuse to give the full body length. The product of sin θ and body length yields an estimate for bipedal MFH.

We took measurements from the literature when they could not be made directly. Examples of complete, articulated vertebral columns from the DPF are nonexistent. For this reason, we assumed that mounts that included restored vertebrae were reasonably accurate and were included in this study; however, entirely missing or restored sections of the vertebral column (e.g., cervicals, thoracics, caudals) or limb elements were not used. We estimated these missing elements from more complete specimens using reduced major axis regression (RMA; Additional file 2). RMA is preferable to ordinary least squares (OLS) regression because it does not assume that the independent variable is measured without error. There is some debate, however, about whether RMA is preferable to OLS when extrapolating beyond the dataset [63], as we occasionally did here. For species lacking postcranial material, we modeled MFHs after closely related taxa of similar size (e.g., *Unescoceratops koppelhusae* was modeled after *Leptoceratops gracilis* [CMN 8889], “*Stegoceras”* (“*Prenocephale*”) *breve* and the unnamed pachycephalosaur after *S. validum* [UALVP 2], and cf. *Orodromeus* after *Parksosaurus warreni* [ROM 802]).

The various sources of error associated with the reconstruction of MFHs must be noted. For instance, habitual limb flexion in any of the taxa considered here would result in overestimated MFHs. Ceratopsids, in particular, are generally thought to have held their forelimbs in a semi-sprawled posture [64-68], which would result in smaller MFHs. Conversely, our inability to account for the influence of epiphyseal cartilage on feeding height calculations would result in underestimated MFHs. Holliday et al. [69] recently showed that, among archosaurs, epiphyseal cartilage significantly increases limb length relative to that calculated from the bones alone, the results differing by up to 10%. By accounting for missing cartilage in multi-tonned dinosaurs using various “cartilage correction factors” (CCFs), Holliday et al. [69] found that the heights of these animals may be consistently underestimated by up to 0.5 m. Unfortunately, it is not possible to determine exactly how much epiphyseal cartilage is missing from every individual, and to apply different CCFs to separate taxa in the reconstruction of maximum feeding heights would tell us more about the influence of CCFs in producing (or reducing) statistical differences, and less about how the fossil bones themselves differ. On the other hand, it is unlikely that the same CCF should apply across all taxa. In the best case scenario, the confounding effects of limb flexion and epiphyseal cartilage on the calculation of maximum feeding heights would cancel each other out. Overall, the error in reconstructing MFH is likely on the decimetre scale. Although this may affect the results in some cases (e.g., discerning between low-level browsers), we believe that this level of error should permit the major patterns to be discerned.

### Statistical comparisons

We compared MFHs at coarse (family/suborder), medium (subfamily/family), and fine (genus) taxonomic scales. We did not consider the species level because sample size was generally too low at this resolution to permit meaningful statistical comparisons. We used the non-parametric Kruskal-Wallis test because samples were typically quite small (n < 30) and non-normal. This test lacks the power of parametric tests, and is more prone to committing Type II errors (reporting false negatives), but is more robust to committing Type I errors (reporting false positives). We conducted posthoc pairwise comparisons using Mann–Whitney U tests with Bonferroni correction. Bonferroni correction was designed to counteract the problem of multiple comparisons, wherein the probability of committing a Type I error increases with the number of simultaneous comparisons being made [70]. This problem is rectified by multiplying the *p*-value by the number of pairwise comparisons, effectively lowering the significance level. However, because Bonferroni correction provides little power and is probably too conservative [70,71], we also report uncorrected probabilities for the purpose of interpretation. We performed all statistical procedures using the software program PAST 2.12 [72].

Because the DPF does not represent a single assemblage of contemporaneous organisms, time-averaging is an issue. This has the effect of masking palaeoecological patterns that are otherwise distinguishable only at fine temporal resolutions [73]. For this reason, we minimized the effects of time-averaging by making the above comparisons within each of the two most inclusive Megaherbivore Assemblage Zones (MAZs) identified by Mallon et al. [51]. To summarize, MAZ-1 encompasses the lower 28 m of the DPF, and MAZ-2 encompasses intervals from 29–52 m. Although this time-constrained approach theoretically increases the probability of recovering differences that would otherwise be masked by the effects of time-averaging, there is a trade-off in that sample size (and hence statistical power) is reduced considerably. Also, this approach does not completely remove the effects of time-averaging because the MAZs are themselves time-averaged over a period of approximately 600 Ka [51]. Although we took steps to minimize the amount of time-averaging in this study, the remaining time bias is likely too large to effectively capture true, interacting palaeocommunities. It is possible to reduce the time bias further by dividing the MAZs into approximately 300 Ka sub-zones [51], more closely approximating true palaeocommunities, but the sample size (and resulting statistical power) per sub-zone becomes drastically reduced in doing so.

## Results

### Time-averaged approach

**Figure 3:**
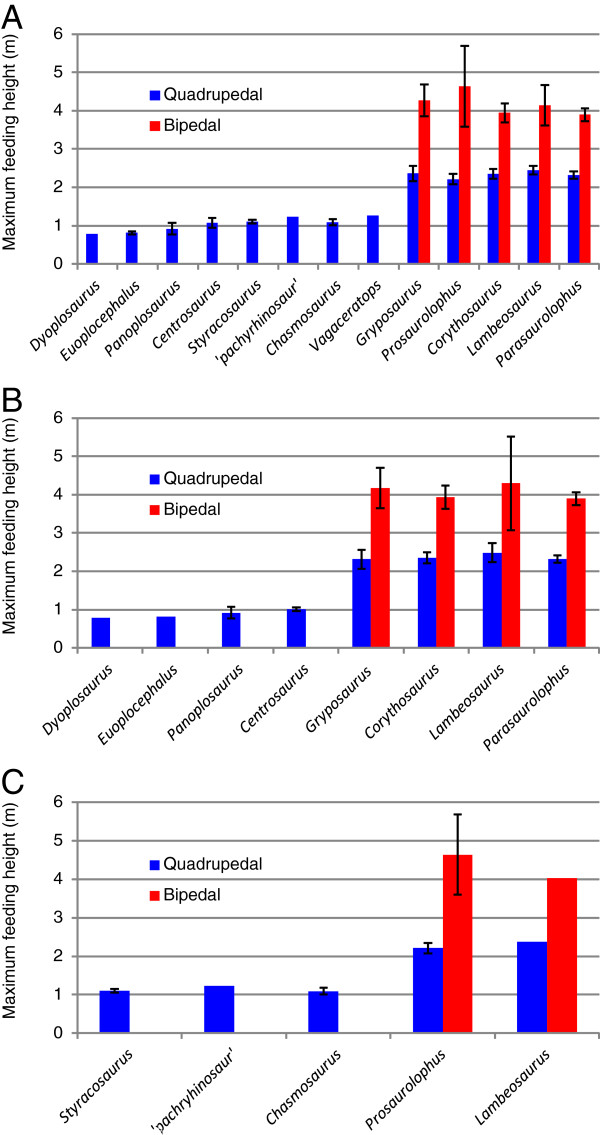
**Reconstructed MFHs of the herbivorous dinosaurs from the DPF**. **A**, time-averaged analysis; **B**, MAZ-1 analysis; **C**, MAZ-2 analysis. Scale bars represent 95% confidence intervals.

The MFHs for the time-averaged analysis are depicted in Figure 3A. The most striking aspect of this diagram is that, irrespective of body mass, most species were restricted to feeding below approximately 1 m from the ground. The leptoceratopsid *Unescoceratops* appears to have been particularly limited in its feeding range because it was unable to reach above 0.5 m while in a quadrupedal posture. However, this animal probably could have reared bipedally to reach somewhat beyond 1 m in a manner similar to other coexisting, small ornithischians (cf. *Orodromeus* and pachycephalosaurids).

With respect to the megaherbivorous forms, the average quadrupedal MFHs of ankylosaurs generally fall slightly below 1 m, whereas those of ceratopsids plot slightly above 1 m. Hadrosaurids clearly have the most distinct MFHs, which consistently plot above 2 m in a quadrupedal posture, or 4 m in a bipedal posture. Hadrosaurines (*Gryposaurus* and *Prosaurolophus*) have slightly taller average bipedal MFHs than lambeosaurines, but the uncertainty intervals for all hadrosaurids in this posture are quite large and overlap substantially. This may reflect variation in reconstructed portions of the spinal column and estimated missing elements.

The Kruskal-Wallis test reveals highly significant differences among all higher-level taxa with adequate representation (N = 62, H = 55.82, *p* < 0.001); Ankylosauria, Ceratopsidae, and Hadrosauridae each differ significantly from one another in quadrupedal MFH (Table 1). As expected, the differences are exacerbated when bipedal hadrosaurids are considered, which differ significantly from all quadrupedal postures. No further significant differences are recovered with increasingly finer taxonomic resolution, even between the apparently different bipedal hadrosaurines and lambeosaurines (Tables 2–3).

**Table 1:** Coarse-scale Mann–Whitney pairwise comparisons of the time-averaged analysis

	**Ankylosauria**	**Ceratopsidae**	**Hadrosauridae (Q)**	**Hadrosauridae (B)**
Ankylosauria		**0.00**	**0.00**	**0.00**
Ceratopsidae	**0.00**		**0.00**	**0.00**
Hadrosauridae (Q)	**0.00**	**0.00**		**0.00**
Hadrosauridae (B)	**0.00**	**0.00**	**0.00**	

N = 62, H = 55.82, *p* = 4.554 × 10^-12^.

Bonferroni corrected *p*-values shown in lower left triangle; uncorrected pairwise comparison *p*-values shown in upper right triangle. Significant results reported in bold. Abbreviations: Q, quadrupedal posture; B, bipedal posture.

**Table 2:** Medium-scale Mann–Whitney pairwise comparisons of the time-averaged analysis

	**An**	**No**	**Ce**	**Ch**	**Ha (Q)**	**Ha (B)**	**La (Q)**	**La (B)**
An		0.51	**0.00**	**0.01**	**0.00**	**0.00**	**0.00**	**0.00**
No	1		0.11	0.19	**0.01**	**0.01**	**0.00**	**0.00**
Ce	**0.04**	1		0.67	**0.00**	**0.00**	**0.00**	**0.00**
Ch	0.30	1	1		**0.01**	**0.01**	**0.00**	**0.00**
Ha (Q)	0.10	0.40	0.07	0.40		**0.00**	0.36	**0.00**
Ha (B)	0.06	0.30	**0.04**	0.30	0.10		**0.00**	0.30
La (Q)	**0.01**	0.11	**0.01**	0.11	1	**0.01**		**0.00**
La (B)	**0.01**	0.11	**0.01**	0.11	**0.02**	1	**0.00**	

N = 62, H = 56.17, *p* = 8.721 × 10^-10^.

Bonferroni corrected *p*-values shown in lower left triangle; uncorrected pairwise comparison *p*-values shown in upper right triangle. Significant results reported in bold. Taxonomic abbreviations: An, Ankylosauridae; Ce, Centrosaurinae; Ch, Chasmosaurinae; Ha, Hadrosaurinae; La, Lambeosaurinae; No, Nodosauridae. Other abbreviations as in Table 1.

**Table 3:** Fine-scale Mann–Whitney pairwise comparisons of the time-averaged analysis

	**Euo**	**Pan**	**Cen**	**Sty**	**Cha**	**Gry (Q)**	**Gry (B)**	**Pro (Q)**	**Pro (B)**	**Cor (Q)**	**Cor (B)**	**Lam (Q)**	**Lam(B)**	**Par(Q)**	**Par(B)**
Euo		0.59	**0.01**	0.07	**0.03**	**0.01**	**0.01**	0.07	**0.03**	**0.00**	**0.00**	**0.01**	**0.01**	0.07	0.07
Pan	1		0.39	0.11	0.38	**0.03**	**0.03**	0.11	**0.05**	**0.01**	**0.01**	**0.03**	**0.03**	0.11	0.11
Cen	0.85	1		0.33	0.55	**0.02**	**0.02**	0.08	**0.04**	**0.01**	**0.01**	**0.02**	**0.02**	0.08	0.08
Sty	1	1	1		0.77	0.11	0.11	0.25	0.15	0.06	0.06	0.11	0.11	0.25	0.25
Cha	1	1	1	1		**0.05**	**0.05**	0.15	0.08	**0.02**	**0.02**	**0.05**	**0.05**	0.15	0.15
Gry (Q)	1	1	1	1	1		**0.03**	0.82	**0.05**	0.92	**0.01**	0.67	**0.03**	0.82	0.11
Gry (B)	1	1	1	1	1	1		0.11	0.60	**0.01**	0.51	**0.03**	0.89	0.11	0.49
Pro (Q)	1	1	1	1	1	1	1		0.15	0.19	0.06	0.11	0.11	0.70	0.25
Pro (B)	1	1	1	1	1	1	1	1		**0.02**	0.36	**0.05**	0.86	0.15	0.77
Cor (Q)	0.36	1	0.61	1	1	1	1	1	1		**0.00**	0.51	**0.01**	0.31	0.06
Cor (B)	0.36	1	0.61	1	1	1	1	1	1	0.23		**0.01**	0.92	0.06	0.38
Lam (Q)	1	1	1	1	1	1	1	1	1	1	1		**0.03**	0.25	0.11
Lam (B)	1	1	1	1	1	1	1	1	1	1	1	1		0.11	0.82
Par (Q)	1	1	1	1	1	1	1	1	1	1	1	1	1		0.25
Par (B)	1	1	1	1	1	1	1	1	1	1	1	1	1	1	

N = 59, H = 53.52, *p* = 1.553 × 10^-6^.

Bonferroni corrected *p*-values shown in lower left triangle; uncorrected pairwise comparison *p*-values shown in upper right triangle. Significant results reported in bold. Taxonomic abbreviations: Cen, *Centrosaurus*; Cha, *Chasmosaurus*; Cor, *Corythosaurus*; Euo, *Euoplocephalus*; Gry, *Gryposaurus*; Lam, *Lambeosaurus*; Pan, *Panoplosaurus*; Par, *Parasaurolophus*; Pro, *Prosaurolophus*; Sty, *Styracosaurus.* Other abbreviations as in Table 1.

### Time-constrained approach

The results of the time-constrained analyses (MAZ-1 and −2) largely mirror those of the time-averaged analysis; the same MFH relationships are maintained (Figure 3B, C). Note that some taxa, while known from the DPF, are not represented in either MAZ-1 or −2 due either to a lack of appropriate material or imprecise provenance data (e.g., ankylosaurs in MAZ-2, *Chasmosaurus* in MAZ-1). Probabilities are reduced in the time-constrained analyses, often below significant levels, due to correspondingly reduced sample sizes (Tables 4, 5, 6, 7, 8 and 9).

**Table 4:** Coarse-scale Mann–Whitney pairwise comparisons of the MAZ-1 analysis

	**Ankylosauria**	**Ceratopsidae**	**Hadrosauridae (Q)**	**Hadrosauridae (B)**
Ankylosauria		0.24	**0.00**	**0.00**
Ceratopsidae	1		**0.00**	**0.00**
Hadrosauridae (Q)	**0.00**	**0.02**		**0.00**
Hadrosauridae (B)	**0.00**	**0.02**	**0.00**	

N = 36, H = 31.11, *p* = 8.049 × 10^-7^.

Bonferroni corrected *p*-values shown in lower left triangle; uncorrected pairwise comparison *p*-values shown in upper right triangle. Significant results reported in bold. Abbreviations as in Table 1.

**Table 5:** Medium-scale Mann–Whitney pairwise comparisons of the MAZ-1 analysis

	**An**	**No**	**Ce**	**Ha (Q)**	**Ha (B)**	**La (Q)**	**La (B)**
An		0.82	0.11	0.15	0.15	**0.04**	**0.04**
No	1		0.67	**0.05**	**0.05**	**0.01**	**0.01**
Ce	1	1		**0.05**	**0.05**	**0.01**	**0.01**
Ha (Q)	1	1	1		0.08	0.55	**0.01**
Ha (B)	1	1	1	1		**0.01**	0.80
La (Q)	0.87	0.12	0.12	1	0.30		**0.00**
La (B)	0.87	0.12	0.12	0.30	1	**0.00**	

N = 36, H = 31.25, *p* = 2.269 × 10^-5^.

Bonferroni corrected *p*-values shown in lower left triangle; uncorrected pairwise comparison *p*-values shown in upper right triangle. Significant results reported in bold. Abbreviations as in Tables 1 and 2.

**Table 6:** Fines-scale Mann–Whitney pairwise comparisons of the MAZ-1 analysis

	**Pan**	**Cen**	**Gry (Q)**	**Gry (B)**	**Cor (Q)**	**Cor (B)**	**Lam (Q)**	**Lam (B)**	**Par (Q)**	**Par (B)**
Pan		0.67	**0.05**	**0.05**	**0.01**	**0.01**	0.11	0.11	0.11	0.11
Cen	1		**0.05**	**0.05**	**0.01**	**0.01**	0.11	0.11	0.11	0.11
Gry (Q)	1	1		0.08	0.90	**0.03**	0.39	0.15	0.77	0.15
Gry (B)	1	1	1		**0.03**	0.90	0.15	0.77	0.15	0.77
Cor (Q)	0.64	0.64	1	1		**0.01**	0.62	0.07	0.40	0.07
Cor (B)	0.64	0.64	1	1	0.23		0.07	0.87	0.07	0.40
Lam (Q)	1	1	1	1	1	1		0.25	0.70	0.25
Lam (B)	1	1	1	1	1	1	1		0.25	0.70
Par (Q)	1	1	1	1	1	1	1	1		0.25
Par (B)	1	1	1	1	1	1	1	1	1	

N = 34, H = 29.26, *p* = 5.86 × 10^-4^.

Bonferroni corrected *p*-values shown in lower left triangle; uncorrected pairwise comparison *p*-values shown in upper right triangle. Significant results reported in bold. Abbrevations as in Tables 1 and 3.

**Table 7:** Coarse-scale Mann–Whitney pairwise comparisons of the MAZ-2 analysis

	**Ceratopsidae**	**Hadrosauridae (Q)**	**Hadrosauridae (B)**
Ceratopsidae		**0.03**	**0.01**
Hadrosauridae (Q)	0.08		**0.05**
Hadrosauridae (B)	**0.04**	0.16	

N = 13, H = 10.38, *p* = 5.559 × 10^-3^.

Bonferroni corrected *p*-values shown in lower left triangle; uncorrected pairwise comparison *p*-values shown in upper right triangle. Significant results reported in bold. Abbreviations as in Table 1.

**Table 8:** Medium-scale Mann–Whitney pairwise comparisons of the MAZ-2 analysis

	**Ce**	**Ch**	**Ha (Q)**	**Ha (B)**
Ce		0.38	0.15	0.08
Ch	1		0.15	0.08
Ha (Q)	0.89	0.89		0.15
Ha (B)	0.49	0.49	0.89	

N = 11, H = 8.561, *p* = 0.03574.

Bonferroni corrected *p*-values shown in lower left triangle; uncorrected pairwise comparison *p*-values shown in upper right triangle. Significant results reported in bold. Abbreviations as in Tables 1 and 2.

**Table 9:** Fine-scale Mann–Whitney pairwise comparisons of the MAZ-2 analysis

	**Sty**	**Cha**	**Pro (Q)**	**Pro (B)**
Sty		0.77	0.25	0.15
Cha	1		0.15	0.08
Pro (Q)	1	0.89		0.15
Pro (B)	0.89	0.49	0.89	

N = 10, H = 7.72,7 *p* = 0.052.

Bonferroni corrected *p*-values shown in lower left triangle; uncorrected pairwise comparison *p*-values shown in upper right triangle. Significant results reported in bold. Abbreviations as in Tables 1 and 3.

## Discussion

### Feeding height stratification in extant ecosystems

Ecological separation of coexisting species is achieved along the axes of food, time, and space [74,75]. For example, the African savannah biome supports 31 species of large (> 5 kg), herbivorous mammals [76], and competition among its members is typically alleviated via the selection of different food types, the occupation of the same habitat at different times, or the occupation of different habitats at the same time [77-79]. The ecological separation of these ungulates may also be achieved along a vertical gradient, with different species feeding at different heights within the canopy or grass cover [77-83]. For example, Bell [78] demonstrated that, among grazers, zebra (*Equus burchelli*) tend to ingest the tallest, most fibrous portions of the herb layer, wildebeest (*Connochaetes taurinus*) and topi (*Damaliscus korrigum*) select the more nutritious middle layer, and Thomson’s gazelle (*Gazella thomsoni*) take in fruits from the ground. With the exception of the topi, this grazing succession is reflected by the decreasing body size of the animals (but see [83]). Similarly, feeding height stratification has been said to operate among browsing ungulates [77,79,81]. This hypothesis was tested explicitly on a subset of African browsers by du Toit [82], who found that giraffe (*Giraffa camelopardalis*), kudu (*Tragelaphus strepsiceras*), impala (*Aepyceros melampus*), and steenbok (*Raphicerus campesteris*) are stratified by mean feeding-height. On average, giraffe feed at heights between 2 and 3 m, kudu at heights near 1 m, and impala and steenbok at heights below 0.5 m. Although there was significant overlap between the browsing heights of some of these species, du Toit [82] observed that feeding height stratification is more pronounced during the dry season, when the use of woody browse is increased to compensate for the reduced availability of green forage in the herb layer. The long neck of the giraffe has been cited as an example of an adaptation to escape competition occurring at lower browsing heights [84].

### Feeding height stratification in the Dinosaur Park Formation assemblage

As documented in extant taxa, numerous studies have commented on the importance of feeding height stratification as a mechanism for niche partitioning among herbivorous dinosaurs. For example, within the Upper Jurassic Morrison Formation of the western United States, up to five sauropod genera are thought to have lived in sympatry [85]. Feeding height stratification has been repeatedly invoked as a means of facilitating their coexistence, with different sauropods using their long necks to feed at different heights within the environment. Evidence for this interpretation includes reconstructed neck morphology [33,34,56,57], tooth wear analysis [44,53-55,58,86], and jaw mechanics [52,54].

Feeding height stratification has likewise been invoked to account for the diverse herbivorous dinosaur fauna of the DPF [12,13,33], but no formal test of this hypothesis has been conducted to date. Notably, most treatments of feeding height stratification make little or no mention of the small ornithischians that inhabited the Late Cretaceous landscape of Laramidia. This is probably because these forms are both poorly known and relatively rare in the fossil record. However, Brown et al. [48] recently demonstrated a substantial taphonomic size bias in the record of the DPF, and it is therefore likely that small ornithischians comprised a larger proportion of the herbivore fauna than previously assumed. The MFHs of these animals were probably restricted to less than approximately 1 m, which may have placed them in competition with the megaherbivores discussed below.

### Ankylosaurs

Béland and Russell [12] and Coe et al. [13] suggested that ankylosaurs from the DPF exhibited different MFHs, with *Euoplocephalus* feeding on herbs below 0.5 m and *Panoplosaurus* feeding on woody vegetation up to 1 m. However, the current data do not support this hypothesis. The mean MFH of *Euoplocephalus* is closer to 0.8 m, whereas that of *Panoplosaurus* approximates 0.9 m. There is also considerable overlap of MFH between these taxa, with some specimens of *Euoplocephalus* reaching as high as 0.88 m, and some specimens of *Panoplosaurus* only reaching 0.77 m. Apart from the reduced ankylosaur sample (see below), this moderate overlap of MFHs likely accounts for the fact that *Euoplocephalus* and *Panoplosaurus* do not differ significantly from one another. The contention of Weishampel and Norman [87] that ankylosaurs were generally restricted to browsing between 1 and 2 m is not supported here. By extending the forelimb proportions of *Euoplocephalus* (AMNH 5403) to the 542 mm-long humerus of *Ankylosaurus* (AMNH 5214), the largest known ankylosaur, the total forelimb length is estimated to be only 1.1 m.

### Ceratopsids

Several authors [12,13,87,88] have likewise suggested that ceratopsids were capable of reaching heights of 2 m; however, no evidence was provided for this value. Instead, our results indicate that ceratopsids more likely browsed no higher than approximately 1 m, as suggested by Dodson [7]. One well-preserved specimen of *Triceratops* (NSM PV 20379), among the largest ceratopsids that ever existed, could not reach above 1.2 m, either [68].

### Hadrosaurids

The common claim that hadrosaurids could reach heights up to 4 m [7,12,13,33,87,88] is supported by our results. The largest hadrosaurids from the DPF, *Gryposaurus* and *Prosaurolophus*, probably could reach heights approaching 5 m in a bipedal posture (Figure 3). Despite these maxima, some authors [87,89] have proposed that hadrosaurid feeding was probably concentrated below 2 m, which would accord with the quadrupedal feeding postures calculated here.

Both vertebrate microfossil and skeletal remains suggest that hadrosaurids formed approximately 40% of the herbivorous dinosaur assemblage in the DPF ecosystem [7,61], which likely translates to a greater proportion of the herbivore biomass because hadrosaurids are the largest members of the fauna. The remainder of herbivores, notably the large ceratopsids and ankylosaurs which combined form approximately an equivalent proportion in terms of relative abundance, fed at or below the 1 m mark. Although feeding heights could not be discriminated below 1 m, the fact that hadrosaurids could reach up to 5 m and were therefore segregated from all other herbivores is likely fundamental in partitioning the resource base. Importantly, hadrosaurids were capable of reaching shrubs and low-growing trees that were beyond the reach of ceratopsids, ankylosaurs, and other small herbivores, effectively dividing the herbivores in terms of relative abundance. This may also have allowed hadrosaurids to escape resource stresses imparted by low browsers, and may have facilitated the co-existence of large herds of ceratopsids and highly abundant hadrosaurids [20,90] in DPF palaeoecosystems.

### Dinosaur browsing and vegetation structure

The prevailing climate of the DPF has been described as warm temperate, as revealed by tree-, tooth-, and bone-growth ring data [91,92], sedimentological data [93], and biogeographic data [94,95]. Regional leaf physiognomic data have also reinforced this interpretation [96]. This climate is, in part, thought to have given rise to both open and closed habitats [96-99] akin to those of modern ecosystems [36]. Braman and Koppelhus ([100]:124) describe the landscape of the DPF as having been “wet everywhere, at least for portions of the year”, with dense vegetation lining the rivers, and more open habitats occurring further distally.

The open habitats of the DPF and surrounding regions were likely dominated by ferns and low-growing angiosperms. Coe et al. ([13]: 235) even proposed the existence of extensive “fern prairies”, analogous to modern grasslands, but Tiffney [101] stressed that evidence for such fern-dominated communities is lacking. Nonetheless, Wing et al. [102] subsequently reported one exceptional fossil flora from the mid-Maastrichtian in which ferns and other “pteridophytes” account for nearly 50% of the total ground cover. By comparison, these same plants account for approximately 40% of the total palynomorph abundance in the DPF [103].

The Late Cretaceous saw the radiation of the angiosperms, which typically took the form of “weedy” herbs and shrubs growing in open or marginal habitats [12,33,97,98,101,104,105]. Angiosperm trees, although inferred to have existed elsewhere [96], probably did not occur in the DPF, as evidenced by the lack of diagnostic fossil wood [100]. It is commonly argued ([98]:125[104,106]) that angiosperms occurred most regularly in coastal and fluvial depositional settings, occupying “stream-side and aquatic habitats, the forest understory and early successional thickets”; however, Wheeler and Lehman [107] noted the existence of angiosperm-dominated communities in southern upland environments as well, where conifers were otherwise thought to have dominated [98,104]. By virtue of their r-selected life history strategies, it is likely that angiosperms were capable of growing in a wide variety of habitats [101].

A common theme in the literature is the persistence of open-habitat cycadophytes (bennettitaleans and cycads) as forage for Late Cretaceous herbivores [34,97,108-113]. However, bennettitaleans went extinct by the Santonian [114], and cycads were probably absent from the DPF ([65,115,116], D. R. Braman, pers. comm., 2012), having been replaced by angiosperms [98,117]. It is by no means clear that cycadophytes were common enough elsewhere during the Late Cretaceous to support dense megaherbivore populations, but their seed coats may have served to supplement dinosaur diets [97,109-111].

Unlike tropical forests, temperate forests typically exhibit limited stratification [118], and there is little reason to suspect that the temperate forests of the DPF were any different. Wolfe and Upchurch [96] proposed that such forests were, in fact, relatively sparse, with sunlight often penetrating fully through to the ground. Palynofloral and macroplant evidence from the DPF suggests that the forest canopy was formed primarily by taxodiaceous, cupressaceous, and podocarpaceous conifers [100,119,120], a composition typical of most Late Cretaceous warm temperate forests [117]. Angiosperm shrubs may [98] or may not [121]) have formed an understory, alongside tree ferns and gymnosperm saplings [100]. The herb layer would have included ferns, lycopods, angiosperm herbs, and gymnosperm saplings, and ground cover comprised mosses, lichens, fungi, hornworts, and decaying vegetable matter [100].

Opinions vary about the degree to which habitat structure influenced the regional distribution of the Late Cretaceous herbivorous dinosaurs. Some [97,101,112] argued that the megaherbivorous forms were likely restricted to feeding in open habitats, partly as a result of their large sizes. However, it appears that the forests of the Late Cretaceous were not particularly dense [96], and probably did not inhibit the movement of even the more massive herbivores [13,96]. Alternatively, Baszio [122] suggested that, within the DPF, ankylosaurs and ceratopsids occupied open habitats, whereas hadrosaurids lived in forested environments. His reasoning was that ankylosaurs and ceratopsids, being limited in their range of vertical movements, could not have taken full advantage of stratified forest vegetation in the same way that hadrosaurids presumably could. However, it is unlikely that hadrosaurids could have accessed the entire forest structure; the canopy was almost certainly out of reach, particularly if Late Cretaceous taxodiaceous and cupressaceous conifers grew as tall as their modern descendants (> 90 m). Hadrosaurids likely could forage among the shrubs of the forest understory, but shrubs were abundant in more open habitats as well [12,33,97,98,101,105]. In that case, there is little reason to suspect that hadrosaurids could not have occupied both open and closed habitats [36], alongside ankylosaurs and ceratopsids.

This does not contradict the idea that certain groups may have preferred certain environments over others. Various lines of sedimentological evidence have been brought to bear on the matter [7,12,60,61,91,123,124]. There simply does not appear to have been any major structural obstacles to impede the movement of these animals. Consider that elephants, which are comparable in size to the megaherbivorous dinosaurs considered here, regularly occupy even dense forests and thickets in search of food [125]. In fact, their movements and feeding habits typically result in the creation of new, more navigable habitats [126,127].

Regardless of where hadrosaurids spent most of their time, it is likely that they usually foraged quadrupedally on abundant, low-lying herbage [87,89,128], occasionally rearing up onto their hindlimbs to feed among the angiosperm shrubs. Additional evidence for bipedal feeding in these animals comes from the Campanian aged Blackhawk Formation of Utah, where hundreds of dinosaur footprints are preserved in association with taxodiaceous conifer and palm roots and fallen logs [129]. In many places, pes prints attributed to hadrosaurids are found straddling the roots. The fact that manus prints are not also found in these areas suggests that these animals were rearing up to feed on the high foliage. This bipedal feeding behaviour would have been particularly beneficial in instances where large herds of low-browsing ceratopsids were passing through the same area [17,20-22]. Dietary niche partitioning could have been achieved among hadrosaurids if they utilized different levels within the shrub layer, as do living ruminants [82]. This may also have served to limit niche overlap between different ontogenetic stages of the same species [130]. The larger feeding heights of the hadrosaurids suggest that these animals were able to reach a wider variety of plant types than other sympatric herbivores. Circumstantial evidence for diet in these animals comes from multiple examples of fossil gut contents [131-133], which preserve conifer and angiosperm browse, including twigs and stems, bark, seeds, leaves, and fruit. Probable hadrosaurid coprolites [134,135] also contain abundant fungally-degraded conifer wood, which would presumably indicate that hadrosaurids fed at ground level at least occasionally. However, in light of the problems associated with the attribution of some of these fossils [32,132,136,137], their interpretation as dietary residues must be regarded with due caution.

Ankylosaurs, ceratopsids, and small ornithischians may have partitioned the herb layer by feeding height, as do the ungulates of the Serengeti today [78]. Ceratopsids, being slightly taller, may have even facilitated the existence of the other forms by cropping the herb layer to expose new growth. Of course, this is a highly speculative scenario requiring further investigation. Unfortunately, no ceratopsid gut contents are known by which to gauge these ideas, but an ankylosaurid cololite from Australia is reported to contain fibrous tissue (probably leaves), angiosperm fruits or endocarps, small seeds, and possible fern sporangia [138]. Ankylosaurs, it would seem, consumed less woody browse than hadrosaurids, which is in line with the interpretation given here.

One final aspect of herbivorous dinosaur ecology bears consideration. Elephants are known to regularly fell trees up to 10 m tall to feed on the otherwise unreachable browse, effectively increasing the feeding envelope of these animals up to three times [13,125]. It is possible that the megaherbivores of the DPF were capable of the same behaviour [13,65]. If so, tree felling may have served to increase dietary overlap between these animals, with the squat ankylosaurs and ceratopsids consuming foliage otherwise in reach of hadrosaurids alone. Unfortunately, while tree felling behaviour among dinosaurs is plausible, there is not yet any evidence supporting this speculation. Similarly, scenarios involving hypsilophodontids and pachycephalosaurids climbing trees to increase their feeding heights [122], while not impossible, are implausible owing to a lack of appropriate skeletal adaptation [139,140]. For these reasons, such highly speculative behaviours are not considered further.

### Evolutionary palaeoecological implications

There is no convincing evidence that feeding height stratification, as revealed by reconstructed MFH, played as significant a role in facilitating herbivorous dinosaur niche partitioning in the DPF as previously assumed [12,13]. Despite the 18 genera considered here—six or more of which typically coexisted at a time [51]—only four statistically distinct MFHs are detected. If niche partitioning did allow herbivorous dinosaurs from the DPF to coexist, it may have been achieved by other means in addition to feeding height stratification. Although this hypothesis has yet to be subjected to rigorous testing, multiple morphological features have been proposed to have fostered the coexistence of these herbivores. For example, Carpenter [38-41] suggested that differences between the tooth and beak shapes of ankylosaurids and nodosaurids may have allowed these taxa to specialize on different plant types. Similarly, differences between centrosaurines and chasmosaurines in cranial [37], mandibular [45], and beak [8,43] morphology have been cited as evidence for dietary niche partitioning. Finally, variations in beak shape [7,34,36,44], tooth morphology [36,46], and skeletal proportions [36] are thought to have enabled hadrosaurines and lambeosaurines to forage differentially. Many of these assumptions have not been tested and require further examination, particularly in light of questions regarding the significance of intraspecific variation and the influence of time-averaging.

The disappearance of ankylosaurs from the upper intervals of MAZ-2 of the DPF [51] suggests the possibility that some change in their habitat structure caused their displacement. Although it is by no means obvious whether such a change did occur, the gradual transgression of the Western Interior Seaway over the approximately 1.5 Ma span of the DPF undoubtedly would have had some influence on the palaeoflora. It may be that some of the herbaceous plants preferred by the ankylosaurs disappeared, but this scenario is difficult to test at present.

Overall, the distribution of herbivore MFHs changed minimally over the course of the DPF. Rather, MFHs were quite stable in spite of rapid and continual species turnover, and roughly the same ratio of low to high browsers was upheld (Figure 3B,C). This, in turn, suggests that time-averaging does not completely obscure palaeoecological signals within the DPF, other than to artificially inflate estimates of standing crop biodiversity. It also suggests that the MFHs maintained by their respective species were evolutionarily stable strategies, and may reflect correlated stability in the growth habits of the surrounding plants. Major changes in habitat structure do not appear to have occurred until the beginning of the Paleocene [101,105], underscoring the importance of low-growing herbage in sustaining Late Cretaceous herbivore faunas.

Finally, it must be noted that, while differences in estimated MFHs are consistent with the hypothesis of feeding height stratification, they are not sufficient for this hypothesis to be true. It is possible that, despite these differences, all herbivore taxa from the DPF spent most of their time feeding at ground level [33,34]. In this sense, they may be compared to grazing ungulates, which spend most of their time feeding on low grasses, despite being physically capable of reaching higher browse. Therefore, although it seems likely from an ecological perspective that the herbivorous dinosaur fauna of the DPF exhibited some form of feeding height stratification, competing hypotheses about the role of this mechanism in the facilitation of niche partitioning are underdetermined [141] by the available evidence. To reject the null hypothesis of no feeding height stratification, it would be necessary to show that the herbivorous dinosaurs browsed to their full potential, utilizing their entire reconstructed MFHs, and did not simply spend all their time feeding at ground level. Unfortunately, this type of behaviour simply does not fossilize. Nevertheless, it may be possible to approximate the amount of time spent feeding at different heights by observing other aspects of morphology. For example, it has been shown that low-level grazers often possess a suite of cranial characteristics that allow them to efficiently crop short grass, including wide, ventrally deflected muzzles, elongate faces, transversely wide paroccipital processes, deep mandibles, and tall withers [142-150]. Similarly, primates feeding close to the ground generally possess narrower dental microwear scratches than those feeding higher up in the forest canopy, a function of mean particle size and the ratio of soil particles to phytoliths [151-153]. These same features might be sought among the herbivorous dinosaurs to more accurately determine their browsing habits.

## Conclusions

Niche partitioning between herbivorous dinosaurs from the DPF could have been facilitated in part by feeding height stratification, as is commonly observed among the ungulates of the Serengeti [78,82]. Small ornithischians (cf. *Orodromeus*, *Unescoceratops*, pachycephalosaurids), ankylosaurs, and ceratopsids were generally restricted to feeding below 1 m, and although there is limited data available for the first group, ankylosaurids and ceratopsids may have partitioned the herb layer. Hadrosaurids may also have fed on herbs, but could have avoided competing with the other forms by feeding on taller angiosperm shrubs in times of duress. Sympatric hadrosaurid species, or ontogenetic stages, also could have avoided competing with one another in this way. There is, as yet, no evidence to suggest that any of these taxa were restricted to feeding in either open or closed habitats.

Despite the evidence for feeding height stratification among ankylosaurs, ceratopsids, and hadrosaurids, there is currently no evidence for feeding height stratification within these clades (with the possible exception of quadrupedal versus bipedal hadrosaurids described above). We note, however, that the problem of underdetermination does not allow us to reject the possibility that some form of feeding height stratification occurred within the herb layer. It is also possible that the coexistence of the herbivorous dinosaurs from the DPF was facilitated by other means in addition to feeding height stratification. The prolonged stability of the palaeoecological relationships identified here supports the contention that the fossil assemblage from the Dinosaur Park Formation constitutes a chronofauna.

### Availability of supporting data

The data sets supporting the results of this article are included within the article (and its additional files).

## Competing interests

The authors declare that they have no competing interests.

## Authors’ contributions

JCM planned the study, gathered the data, ran the statistics, and wrote the manuscript. DCE contributed to planning the study, collecting the data, and writing the manuscript. MJR and JSA contributed to planning the study and writing the manuscript. All authors reviewed the final draft of the manuscript. All authors read and approved the final manuscript.

## Supplementary Material

Additional file 1 Data used in this study. Click here for file

Additional file 2 Reduced major axis regression results used to estimate missing elements in this study. Click here for file

## References

[B1] Archibald JD Dinosaur Extinction and the End of an Era: What the Fossils Say 1996 New York: Columbia University Press

[B2] Weishampel DB Young L Dinosaurs of the East Coast 1996 Baltimore: Johns Hopkins University Press

[B3] Zanno LE Makovicky PJ Herbivorous ecomorphology and specialization patterns in theropod dinosaur evolution Proc Natl Acad Sci 2011 108 232 237 10.1073/pnas.1011924108 21173263 PMC3017133

[B4] Lehman TM Late Maastrichtian paleoenvironments and dinosaur biogeography in the Western Interior of North America Palaeogeogr Palaeoclimatol Palaeoecol 1987 60 189 217

[B5] Lehman TM Wolberg DL, Stump E, Rosenberg GD Late Campanian dinosaur biogeography in the Western Interior of North America Dinofest International: Proceedings of a Symposium Sponsored by Arizona State University 1997 Philadelphia: Academy of Natural Sciences 223 240

[B6] Lehman TM Tanke DH, Carpenter K Late Cretaceous dinosaur provinciality Mesozoic Vertebrate Life 2001 Bloomington: Indiana University Press 310 328

[B7] Dodson P A faunal review of the Judith River (Oldman) Formation, Dinosaur Provincial Park, Alberta Mosasaur 1983 1 89 118

[B8] Sampson SD Loewen MA Ryan MJ, Chinnery-Allgeier BJ, Eberth DA Unraveling a radiation: a review of the diversity, stratigraphic distribution, biogeography, and evolution of horned dinosaurs (Ornithischia: Ceratopsidae) New Perspectives on Horned Dinosaurs: The Royal Tyrrell Museum Ceratopsian Symposium 2010 Bloomington: Indiana University Press 405 427

[B9] Gates TA Sampson SD Zanno LE Roberts EM Eaton JG Nydam RL Hutchison JH Smith JA Loewen MA Getty MA Biogeography of terrestrial and freshwater vertebrates from the Late Cretaceous (Campanian) Western Interior of North America Palaeogeogr Palaeoclimatol Palaeoecol 2010 291 371 387 10.1016/j.palaeo.2010.03.008

[B10] du Toit JT Cumming DHM Functional significance of ungulate diversity in African savannas and the ecological implications of the spread of pastoralism Biodiversity Conserv 1999 8 1643 1661 10.1023/A:1008959721342

[B11] Farlow JO A consideration of the trophic dynamics of a Late Cretaceous large-dinosaur community (Oldman Formation) Ecology 1976 57 841 857 10.2307/1941052

[B12] Béland P Russell DA Paleoecology of Dinosaur Provincial Park (Cretaceous), Alberta, interpreted from the distribution of articulated vertebrate remains Can J Earth Sci 1978 15 1012 1024 10.1139/e78-109

[B13] Coe MJ Dilcher DL Farlow JO Jarzen DM Russell DA Friis EM, Chaloner WG, Crane PR Dinosaurs and land plants The Origins of Angiosperms and their Biological Consequences 1987 Cambridge: Cambridge University Press 225 258

[B14] Farlow JO Dodson P Chinsamy A Dinosaur biology Annu Rev Ecol Syst 1995 26 445 471

[B15] Horner JR Makela R Nest of juveniles provides evidence of family structure among dinosaurs Nature 1979 282 296 298 10.1038/282296a0

[B16] Lockley MG Young BH Carpenter K Hadrosaur locomotion and herding behavior: evidence from footprints in the Mesaverde Formation, Grand Mesa Coal Field, Colorado Mountain Geol 1983 20 5 14

[B17] Currie PJ Dodson P Reif W-E, Westphal F Mass death of a heard of ceratopsian dinosaurs Third Symposium of Mesozoic Terrestrial Ecosystems 1984 Tübingen: Attempto Verlag 52 60

[B18] Varricchio DJ Horner JR Hadrosaurid and lambeosaurid bone beds from the Upper Cretaceous Two Medicine Formation of Montana: taphonomic and biologic implications Can J Earth Sci 1993 30 997 1006 10.1139/e93-083

[B19] Lockley MG Hunt AP Dinosaur Tracks and Other Fossil Footprints of the Western United States 1995 New York: Columbia University Press

[B20] Ryan MJ Russell AP Eberth DA Currie PJ The taphonomy of a *Centrosaurus* (Ornithischia: Ceratopsidae) bone bed from the Dinosaur Park Formation (Upper Campanian), Alberta, Canada, with comments on cranial ontogeny PALAIOS 2001 16 482 506 10.1669/0883-1351(2001)016<0482:TTOACO>2.0.CO;2

[B21] Eberth DA Brinkman DB Barkas V Ryan MJ, Chinnery-Allgeier BJ, Eberth DA A centrosaurine mega-bonebed from the Upper Cretaceous of southern Alberta: implications for behavior and death events Perspectives on Horned Dinosaurs: The Royal Tyrrell Museum Ceratopsian Symposium 2010 Bloomington: Indiana University Press 495 508

[B22] Hunt RK Farke AA Ryan MJ, Chinnery-Allgeier BJ, Eberth DA Behavioral interpretations from ceratopsid bonebeds Perspectives on Horned Dinosaurs: The Royal Tyrrell Museum Ceratopsian Symposium 2010 Bloomington: Indiana University Press 447 455

[B23] Sampson SD Dinosaur Odyssey: Fossil Threads in the Web of Life 2009 Berkeley: University of California Press

[B24] Ostrom JH A reconsideration of the paleoecology of hadrosaurian dinosaurs Am J Sci 1964 262 975 997 10.2475/ajs.262.8.975

[B25] Janis CM Damuth J Theodor JM Miocene ungulates and terrestrial primary productivity: where have all the browsers gone? Proc Natl Acad Sci 2000 97 7899 7904 10.1073/pnas.97.14.7899 10884422 PMC16642

[B26] Janis CM Damuth J Theodor JM The species richness of Miocene browsers, and implications for habitat type and primary productivity in the North American grassland biome Palaeogeogr Palaeoclimatol Palaeoecol 2004 207 371 398 10.1016/j.palaeo.2003.09.032

[B27] Miller C LaPasha C Flora of the Early Cretaceous Kootenai Formation in Montana, conifers Palaeontogr Abt B 1984 193 1 17

[B28] Beerling DJ Modelling palaeophotosynthesis: Late Cretaceous to present Phil. Trans. R. Soc. B 1994 346 421 432 10.1098/rstb.1994.0159

[B29] Beerling DJ Global terrestrial productivity in the Mesozoic era Geol Soc London, Spec Publ 2000 181 17 32 10.1144/GSL.SP.2000.181.01.03

[B30] Decherd SM Primary productivity and forage quality of Ginkgo biloba in response to elevated carbon dioxide and oxygen – an experimental approach to mid-Mesozoic paleoecology PhD thesis 2006: North Carolina State University, Department of Marine, Earth, and Atmospheric Sciences

[B31] Sinclair ARE Does interspecific competition or predation shape the African ungulate community? J Anim Ecol 1985 54 899 918 10.2307/4386

[B32] Sander PM Gee CT Hummel J Clauss M Gee CT Mesozoic plants and dinosaur herbivory Plants in Mesozoic Time: Morphological Innovations, Phylogeny, Ecosystems 2010 Bloomington: Indiana University Press 331 359

[B33] Bakker RT Dinosaur feeding behaviour and the origin of flowering plants Nature 1978 274 661 663 10.1038/274661a0

[B34] Bakker RT The Dinosaur Heresies: New Theories Unlocking the Mystery of the Dinosaurs and their Extinction 1986 New York: Zebra Books

[B35] Dodson P Taxonomic implications of relative growth in lambeosaurine hadrosaurs Syst Biol 1975 24 37 54 10.1093/sysbio/24.1.37

[B36] Carrano MT Janis CM Sepkoski JJ Jr Hadrosaurs as ungulate parallels: lost lifestyles and deficient data Acta Palaeontol Pol 1999 44 237 261

[B37] Henderson DM Ryan MJ, Chinnery Allgeier BJ, Eberth DA Skull shapes as indicators of niche partitioning by sympatric chasmosaurine and centrosaurine dinosaurs Perspectives on Horned Dinosaurs: The Royal Tyrrell Museum Ceratopsian Symposium 2010 Bloomington: Indiana University Press 293 307

[B38] Carpenter K Skeletal and dermal armor reconstructions of *Euoplocephalus tutus* (Ornithischia: Ankylosauridae) from the Late Cretaceous Oldman Formation of Alberta Can J Earth Sci 1982 19 689 697 10.1139/e82-058

[B39] Carpenter K Currie PJ, Padian K Ankylosauria Encyclopedia of Dinosaurs 1997 San Diego: Academic Press 16 20

[B40] Carpenter K Farlow JO, Brett-Surman MK Ankylosaurs The Complete Dinosaur 1997 Bloomington: Indiana University Press 307 316

[B41] Carpenter K Redescription of *Ankylosaurus magniventris* Brown 1908 (Ankylosauridae) from the Upper Cretaceous of the Western Interior of North America Can J Earth Sci 2004 41 961 986 10.1139/e04-043

[B42] Campione NE Evans DC Cranial growth and variation in *Edmontosaurus* (Dinosauria: Hadrosauridae): implications for latest Cretaceous megaherbivore diversity in North America PLoS One 2011 6 9 e25186 10.1371/journal.pone.0025186 21969872 PMC3182183

[B43] Lull RS A revision of the Ceratopsia or horned dinosaurs Mem Peabody Mus Nat Hist 1933 3 1 175

[B44] Whitlock JA Inferences of diplodocoid (Sauropoda: Dinosauria) feeding behavior from snout shape and microwear analyses PLoS One 2011 6 4 e18304 10.1371/journal.pone.0018304 21494685 PMC3071828

[B45] Ostrom JH Functional morphology and evolution of the ceratopsian dinosaurs Evolution 1966 20 290 308 10.2307/2406631 28562975

[B46] Erickson GM Krick BA Hamilton M Bourne GR Norell MA Lilleodden E Sawyer WG Complex dental structure and wear biomechanics in hadrosaurid dinosaurs Science 2012 338 98 101 10.1126/science.1224495 23042891

[B47] Ryan MJ Evans DC Currie PJ, Koppelhus EB Ornithischian dinosaurs Dinosaur Provincial Park: a Spectacular Ancient Ecosystem Revealed 2005 Bloomington: Indiana University Press 312 348

[B48] Brown CM Evans DC Campione NE O’Brien LJ Eberth DA Evidence for taphonomic size bias in a model Mesozoic terrestrial alluvial-paralic system Palaeogeogr Palaeoclimatol Palaeoecol 2013 372 108 122

[B49] Sternberg CM Steveville—West of the Fourth Meridian, Alberta *Geological Survey of Canada Topographic Map 969A*. 1950 Ottawa: Geological Survey of Canada

[B50] Currie PJ Russell DA Currie PJ, Koppelhus EB The geographic and stratigraphic distribution of articulated and associated dinosaur remains Dinosaur Provincial Park: a Spectacular Ancient Ecosystem Revealed 2005 Bloomington: Indiana University Press 537 569

[B51] Mallon JC Evans DC Ryan MJ Anderson JS Megaherbivorous dinosaur turnover in the Dinosaur Park Formation (upper Campanian) of Alberta, Canada Palaeogeogr Palaeoclimatol Palaeoecol 2012 350–352 124 138

[B52] Calvo JO Jaw mechanisms in sauropod dinosaurs Gaia 1994 10 183 194

[B53] Fiorillo AR Kielan-Jaworowska Z, Heintz N, Nakrem HA **Dental microwear on the teeth of*****Camarasaurus*****and*****Diplodocus*****: implications for sauropod paleoecology** Fifth Symposium on Mesozoic Terrestrial Ecosystems and Biota 1991 Oslo: Paleontologisk Museum, University of Oslo 23 24

[B54] Fiorillo AR **Dental microwear patterns of the sauropod dinosaurs*****Camarasaurus*****and*****Diplodocus*****: evidence for resource partitioning in the Late Jurassic of North America** Historical Biol 1998 13 1 16 10.1080/08912969809386568

[B55] Fiorillo AR Sankey JT, Baszio S Lack of variability in feeding patterns of the sauropod dinosaurs *Diplodocus* and *Camarasaurus* (Late Jurassic, western USA) with respect to climate as indicated by tooth wear features Vertebrate Microfossil Assemblages: their Role in Paleoecology and Paleobiogeography 2008 Bloomington: Indiana University Press 104 116

[B56] Stevens KA Parrish JM Neck posture and feeding habits of two Jurassic sauropod dinosaurs Science 1999 284 798 800 10.1126/science.284.5415.798 10221910

[B57] Stevens KA Parrish JM Currie Rogers KA, Wilson JA Digital reconstructions of sauropod dinosaurs and implications for feeding The Sauropods: Evolution and Paleobiology 2005 Berkeley: University of California Press 178 200

[B58] Upchurch P Barrett PM Sues H-D The evolution of sauropod feeding mechanisms Evolution of Herbivory in Terrestrial Vertebrates: Perspectives from the Fossil Record 2000 Cambridge: Cambridge University Press 79 122

[B59] Taylor MP Wedel MJ Naish D Head and neck posture in sauropod dinosaurs inferred from extant animals Acta Palaeontol Pol 2009 54 213 220 10.4202/app.2009.0007

[B60] Brinkman DB Paleoecology of the Judith River Formation (Campanian) of Dinosaur Provincial Park, Alberta, Canada: evidence from vertebrate microfossil localities Palaeogeogr Palaeoclimatol Palaeoecol 1990 78 37 54 10.1016/0031-0182(90)90203-J

[B61] Brinkman DB Ryan MJ Eberth DA The paleogeographic and stratigraphic distribution of ceratopsids (Ornithischia) in the upper Judith River Group of western Canada PALAIOS 1998 13 160 169 10.2307/3515487

[B62] Brown CM Russell AP Homology and architecture of the caudal basket of Pachycephalosauria (Dinosauria: Ornithischia): the first occurrence of myorhabdoi in Tetrapoda PLoS One 2012 7 1 e30212 10.1371/journal.pone.0030212 22272307 PMC3260247

[B63] Smith RJ Use and misuse of the reduced major axis for line-fitting Am J Phys Anthropol 2009 140 476 486 10.1002/ajpa.21090 19425097

[B64] Johnson RE Ostrom JH Thomason JJ The forelimb of *Torosaurus* and an analysis of the posture and gait of ceratopsians Functional Morphology in Vertebrate Paleontology 1995 Cambridge: Cambridge University Press 205 218

[B65] Dodson P The Horned Dinosaurs: a Natural History 1996 Princeton: Princeton University Press

[B66] Dodson P Farlow JO Wolberg DL, Stump E, Rosenberg GD The forelimb carriage of ceratopsid dinosaurs Dinofest International 1997 Philadelphia: Academy of Natural Sciences 393 398

[B67] Thompson S Holmes R Forelimb stance and step cycle in *Chasmosaurus irvinensis* (Dinosauria: Neoceratopsia) Palaeontol Electron 2007 10 1 http://palaeo-electronica.org/paleo/2007_1/step

[B68] Fujiwara S-I A reevaluation of the manus structure in *Triceratops* (Ceratopsia: Ceratopsidae) J Vert Paleontol 2009 29 1136 1147 10.1671/039.029.0406

[B69] Holliday CM Ridgely RC Sedlmayr JC Witmer LM Cartilaginous epiphyses in extant archosaurs and their implications for reconstructing limb function in dinosaurs PLoS One 2010 5 9 e13120 10.1371/journal.pone.0013120 20927347 PMC2948032

[B70] Sokal RR Rohlf FJ Biometry 1995 3 New York: W. H. Freeman and Company

[B71] Nakagawa S A farewell to Bonferroni: the problems of low statistical power and publication bias Behav Ecol 2004 15 1044 1045 10.1093/beheco/arh107

[B72] Hammer Ø Harper DAT Ryan PD PAST: paleontological statistics software package for education and data analysis Palaeontol Electron 2001 4 1 http://palaeo-electronica.org/2001_1/past/issue1_01.htm

[B73] Behrensmeyer AK Hook RW Behrensmeyer AK, Damuth JD, DiMichele WA, Potts R, Sues H-D, Wing SL Paleoenvironmental contexts and taphonomic modes Terrestrial Ecosystems through Time: Evolutionary Paleoecology of Terrestrial Plants and Animals 1992 Chicago: University of Chicago Press 15 136

[B74] Pianka ER The structure of lizard communities Annu Rev Ecol Syst 1973 4 53 74 10.1146/annurev.es.04.110173.000413

[B75] Schoener TW Resource partitioning in ecological communities Science 1974 185 27 39 10.1126/science.185.4145.27 17779277

[B76] Owen-Smith RN Huntley BJ, Walker BH Factors influencing the consumption of plant products by large herbivores Ecology of Tropical Savannas 1982 Berlin: Springer-Verlag 359 404

[B77] Lamprey H Lamprey HF Ecological separation of the large mammal species in the Tarangire Game Reserve, Tanganyika Afr J Ecol 1963 1 63 92 10.1111/j.1365-2028.1963.tb00179.x

[B78] Bell RHV A grazing ecosystem in the Serengeti Sci Am 1971 224 86 935538697

[B79] McNaughton SJ Georgiadis NJ Ecology of African grazing and browsing mammals Annu Rev Ecol Syst 1986 17 39 65 10.1146/annurev.es.17.110186.000351

[B80] Hirst SM Ungulate habitat relationships in a South African woodland/savanna ecosystem Wildlife Monogr 1975 44 1 60

[B81] Leuthold W Ecological separation among browsing ungulates in the Tsavo East National Park, Kenya Oecologia 1978 35 241 252 10.1007/BF00344735 28309736

[B82] du Toit JT Feeding-height stratification among African browsing ruminants Afr J Ecol 1990 28 55 61 10.1111/j.1365-2028.1990.tb01136.x

[B83] Arsenault R Owen-Smith N Resource partitioning by grass height among grazing ungulates does not follow body size relation Oikos 2008 117 1711 1717 10.1111/j.1600-0706.2008.16575.x

[B84] Cameron EZ du Toit JT Winning by a neck: tall giraffes avoid competing with shorter browsers Amer Nat 2007 169 130 135 10.1086/509940 17206591

[B85] Fiorillo AR Time resolution at Carnegie Quarry (Morrison Formation: Dinosaur National Monument, Utah) Univ Wyo Contrib Geol 1994 30 149 156

[B86] Barrett PM Upchurch P Sun A, Wang Y Sauropod feeding mechanisms: their bearing on palaeoecology Sixth Symposium on Mesozoic Terrestrial Ecosystems and Biota, Short Papers 1995 Beijing: China Ocean Press 107 110

[B87] Weishampel DB Norman DB Vertebrate herbivory in the Mesozoic; jaws, plants, and evolutionary metrics Geol Soc Am Spec Pap 1989 238 87 100

[B88] Weishampel DB Jianu C-M Sues H-D Plant-eaters and ghost lineages: dinosaurian herbivory revisited Evolution of Herbivory in Terrestrial Vertebrates: Perspectives from the Fossil Record 2000 Cambridge: Cambridge University Press 123 143

[B89] Horner JR Weishampel DB Forster CA Weishampel DB, Dodson P, Osmólska H Hadrosauridae The Dinosauria 2004 2 Berkeley: University of California Press 438 463

[B90] Eberth DA Evans DC Braman DR, Eberth DA, Evans DC, Taylor W First documented hadrosaurid bonebed from the Belly River Group (Campanian) at Dinosaur Provincial Park, Alberta, Canada: importance and implications International Hadrosaur Symposium at the Royal Tyrrell Museum of Palaeontology, September 22–23, 2011; Drumheller 2011: Royal Tyrrell Museum 45 46

[B91] Dodson P Sedimentology and taphonomy of the Oldman Formation (Campanian), Dinosaur Provincial Park, Alberta (Canada) Palaeogeogr Palaeoclimatol Palaeoecol 1971 10 21 74 10.1016/0031-0182(71)90044-7

[B92] Johnston PA Growth rings in dinosaur teeth Nature 1979 278 635 636 10.1038/278635a0

[B93] Eberth DA Currie PJ, Koppelhus EB The geology Dinosaur Provincial Park: a Spectacular Ancient Ecosystem Revealed 2005 Bloomington: Indiana University Press 54 82

[B94] Brinkman DB A review of nonmarine turtles from the Late Cretaceous of Alberta Can J Earth Sci 2003 40 557 571 10.1139/e02-080

[B95] Brinkman DB Currie PJ, Koppelhus EB Turtles: diversity, paleoecology, and distribution Dinosaur Provincial Park: a Spectacular Ancient Ecosystem Revealed 2005 Bloomington: Indiana University Press 202 220

[B96] Wolfe JA Upchurch GR Jr North American nonmarine climates and vegetation during the Late Cretaceous Palaeogeogr Palaeoclimatol Palaeoecol 1987 61 33 77

[B97] Krassilov VA Changes of Mesozoic vegetation and the extinction of dinosaurs Palaeogeogr Palaeoclimatol Palaeoecol 1981 34 207 224

[B98] Crane PR Friis EM, Chaloner WG, Crane PR Vegetational consequences of the angiosperm diversification The Origins of Angiosperms and their Biological Consequences 1987 Cambridge: Cambridge University Press 107 144

[B99] Upchurch GR Wolfe JA Friis EM, Chaloner WG, Crane PR Mid-Cretaceous to Early Tertiary vegetation and climate: evidence from fossil leaves and woods The Origins of Angiosperms and Their Biological Consequences 1987 Cambridge: Cambridge University Press 75 105

[B100] Braman DR Koppelhus EB Currie PJ, Koppelhus EB Campanian palynomorphs Dinosaur Provincial Park: a Spectacular Ancient Ecosystem Revealed 2005 Bloomington: Indiana University Press 101 130

[B101] Tiffney BH The role of vertebrate herbivory in the evolution of land plants Palaeobotanist 1992 41 87 97

[B102] Wing SL Hickey LJ Swisher CC Implications of an exceptional fossil flora for Late Cretaceous vegetation Nature 1993 363 342 344 10.1038/363342a0

[B103] Jarzen DM Palynology of Dinosaur Provincial Park (Campanian) Alberta Syllogeus 1982 38 1 69

[B104] Retallack GJ Dilcher DL Cretaceous angiosperm invasion of North America Cretaceous Res 1986 7 227 252 10.1016/0195-6671(86)90027-3

[B105] Wing SL Tiffney BH The reciprocal interaction of angiosperm evolution and tetrapod herbivory Rev Palaeobot Palynol 1987 50 179 210 10.1016/0034-6667(87)90045-5

[B106] Upchurch GR Jr Wolfe JA Cretaceous vegetation of the Western Interior and adjacent regions of North America Geol Assoc Can Spec Pap 1993 39 243 281

[B107] Wheeler EA Lehman TM Upper Cretaceous–Paleocene conifer woods from Big Bend National Park, Texas Palaeogeogr Palaeoclimatol Palaeoecol 2005 226 233 258 10.1016/j.palaeo.2005.05.014

[B108] Ostrom JH A functional analysis of jaw mechanics in the dinosaur *Triceratops* Postilla 1964 88 1 35

[B109] Weishampel DB Interactions between Mesozoic plants and vertebrates: fructifications and seed predation N Jb Geol Paläont Abh 1984 167 224 250

[B110] Taggart RE Cross AT Wolberg DL, Stump E, Rosenberg GD The relationship between land plant diversity and productivity and patterns of dinosaur herbivory Dinofest International: Proceedings of a Symposium Sponsored by Arizona State University 1997 Philadelphia: Academy of Natural Sciences 403 416

[B111] Mustoe GE Coevolution of cycads and dinosaurs Cycad Newsl 2007 30 6 9

[B112] Aulenback KR Identification Guide to the Fossil Plants of the Horseshoe Canyon Formation of Drumheller, Alberta 2009 Calgary: University of Calgary Press

[B113] Tanoue K Grandstaff BS You H-L Dodson P Jaw mechanics in basal Ceratopsia (Ornithischia, Dinosauria) Anat Rec 2009 292 1352 1369 10.1002/ar.20979 19711460

[B114] Nichols DJ Johnson KR Plants and the K-T Boundary 2008 Cambridge: Cambridge University Press

[B115] Dodson P Comparative craniology of the Ceratopsia Am J Sci 1993 293A 200 234

[B116] Dodson P Currie PJ, Padian K Neoceratopsia Encyclopedia of Dinosaurs 1997 San Diego: Academic Press 473 478

[B117] Wing SL Sues H-D Behrensmeyer AK, Damuth JD, DiMichele WA, Potts R, Sues H-D, Wing SL Mesozoic and early Cenozoic terrestrial ecosystems Terrestrial Ecosystems through Time: Evolutionary Paleoecology of Terrestrial Plants and Animals 1992 Chicago: University of Chicago Press 327 416

[B118] Allaby M Temperate Forests. Revised edition 2008 New York: Facts on File, Inc

[B119] Ramanujam CGK Fossil coniferous woods from the Oldman Formation (Upper Cretaceous) of Alberta Can J Earth Sci 1972 50 595 602

[B120] Koppelhus EB Currie PJ, Koppelhus EB Paleobotany Dinosaur Provincial Park: a Spectacular Ancient Ecosystem Revealed 2005 Bloomington: Indiana University Press 131 138

[B121] Crabtree DR Angiosperms of the northern Rocky Mountains: Albian to Campanian (Cretaceous) megafossil floras Ann Missouri Bot Gard 1987 74 707 747 10.2307/2399448

[B122] Baszio S Palaeo-ecology of dinosaur assemblages throughout the Late Cretaceous of South Alberta, Canada Courier Forschungsinst Senckenberg 1997 196 1 31

[B123] Retallack GJ Wolberg DL, Stump E, Rosenberg GD Dinosaurs and dirt Dinofest International: Proceedings of a Symposium Sponsored by Arizona State University 1997 Philadelphia: Academy of Natural Sciences 345 359

[B124] Lyson TR Longrich NR Spatial niche partitioning in dinosaurs from the latest Cretaceous (Maastrichtian) of North America Proc R Soc B 2010 278 1158 1164 20943689 10.1098/rspb.2010.1444 PMC3049066

[B125] Owen-Smith RN Megaherbivores: the Influence of very Large Body Size on Ecology 1988 Cambridge: Cambridge University Press

[B126] Ben-Shahar R Patterns of elephant damage to vegetation in northern Botswana Biol Conserv 1993 65 249 256 10.1016/0006-3207(93)90057-8

[B127] Fritz H Duncan P Gordon IJ Illius AW Megaherbivores influence trophic guilds structure in African ungulate communities Oecologia 2002 131 620 625 10.1007/s00442-002-0919-3 28547558

[B128] Dilkes DW An ontogenetic perspective on locomotion in the Late Cretaceous dinosaur *Maiasaura peeblesorum* (Ornithischia: Hadrosauridae) Can J Earth Sci 2001 38 1205 1227 10.1139/e01-016

[B129] Balsley JK Parker LR **Cretaceous wave-dominated delta, barrier islands, and submarine fan depositional systems: Book Cliffs, east-central Utah** Amer Assoc Petrol Geol Field Guide 1983

[B130] Werner EE Gilliam JF The ontogenetic niche and species interactions in size- structured populations Annu Rev Ecol Syst 1984 15 393 425 10.1146/annurev.es.15.110184.002141

[B131] Kräusel R Die Nahrung von *Trachodon* Paläontol Z 1922 4 8023614030

[B132] Currie PJ Koppelhus EB Muhammad AF Sun A, Wang Y Stomach contents of a hadrosaur from the Dinosaur Park Formation (Campanian, Upper Cretaceous) of Alberta, Canada Sixth Symposium on Mesozoic Terrestrial Ecosystems and Biota, Short Papers 1995 Beijing: China Ocean Press 111 114

[B133] Tweet JS Chin K Braman DR Murphey NL Probable gut contents within a specimen of *Brachylophosaurus canadensis* (Dinosauria: Hadrosauridae) from the Upper Cretaceous Judith River Formation of Montana PALAIOS 2008 23 624 635 10.2110/palo.2007.p07-044r

[B134] Chin K Gill BD Dinosaurs, dung beetles, and conifers: participants in a Cretaceous food web PALAIOS 1996 11 280 285 10.2307/3515235

[B135] Chin K The paleobiological implications of herbivorous dinosaur coprolites from the Upper Cretaceous Two Medicine Formation of Montana: why eat wood? PALAIOS 2007 22 554 566 10.2110/palo.2006.p06-087r

[B136] Abel O Diskussion zu den Vorträgen R. Kräusel und F. Versluys Paläont Z 1922 4 8723614030

[B137] Chin K Currie PJ, Padian K Coprolites Encyclopedia of Dinosaurs 1997 San Diego: Academic Press 147 150

[B138] Molnar RE Clifford HT Carpenter K An ankylosaurian cololite from the Lower Cretaceous of Queensland, Australia The Armored Dinosaurs 2001 Bloomington: Indiana University Press 399 412

[B139] Galton PM *Hypsilophodon*, the cursorial nonarboreal dinosaur Nature 1971 231 159 161 10.1038/231159a0 4930672

[B140] Galton PM The mode of life of *Hypsilophodon*, the supposedly arboreal ornithopod dinosaur Lethaia 1971 4 453 465 10.1111/j.1502-3931.1971.tb01866.x

[B141] Turner D Paleontology: a Philosophical Introduction 2011 Cambridge: Cambridge University Press

[B142] Janis CM Ehrhardt D Correlation of relative muzzle width and relative incisor width with dietary preference in ungulates Zool J Linn Soc 1988 92 267 284 10.1111/j.1096-3642.1988.tb01513.x

[B143] Solounias N Dawson-Saunders B Dietary adaptations and paleoecology of the Late Miocene ruminants from Pikermi and Samos in Greece Palaeogeogr Palaeoclimatol Palaeoecol 1988 65 149 172 10.1016/0031-0182(88)90021-1

[B144] Janis CM Correlation of cranial and dental variables with dietary preferences in mammals: a comparison of macropodoids and ungulates Mem Queensl Mus 1990 28 349 366

[B145] Janis CM Thomason JJ Correlations between craniodental morphology and feeding behavior in ungulates: reciprocal illumination between living and fossil taxa Functional Morphology in Vertebrate Paleontology 1995 Cambridge: Cambridge University Press 76 98

[B146] Solounias N Moelleken SMC Dietary adaptation of some extinct ruminants determined by premaxillary shape J Mammal 1993 74 1059 1071 10.2307/1382445

[B147] Spencer LM Morphological correlates of dietary resource partitioning in the African Bovidae J Mammal 1995 76 448 471 10.2307/1382355

[B148] Mendoza M Janis CM Palmqvist P Characterizing complex craniodental patterns related to feeding behaviour in ungulates: a multivariate approach J Zool 2002 258 223 246 10.1017/S0952836902001346

[B149] Fraser D Theodor JM Anterior dentary shape as an indicator of diet in ruminant artiodactyls J Vertebr Paleontol 2011 31 1366 1375 10.1080/02724634.2011.605404

[B150] Fraser D Theodor JM Comparing ungulate dietary proxies using discriminant function analysis J Morphol 2011 272 1513 1526 10.1002/jmor.11001 21915893

[B151] Walker A Wear striations on the incisors of ceropithecid monkeys as an index of diet and habitat preference Am J Phy Anthropol 1976 45 299 308 10.1002/ajpa.1330450215 822732

[B152] Ungar PS Incisor microwear of Sumatran anthropoid primates Am J Phys Anthropol 1994 94 339 363 10.1002/ajpa.1330940305 7943190

[B153] Ungar PS Dental microwear of European Miocene catarrhines: evidence for diets and tooth use J Hum Evol 1996 31 335 366 10.1006/jhev.1996.0065

