# Supplementary material for: Feeding height stratification among the herbivorous dinosaurs from the Dinosaur Park Formation (upper Campanian) of Alberta, Canada
Source: BMC Ecol. 2013 Apr 4;13:14. doi: 10.1186/1472-6785-13-14 (PMC3637170; doi:10.1186/1472-6785-13-14)
Supplement: Additional file 1 — Data used in this study. [file 1472-6785-13-14-S1.doc]

Additional file 1

**Data used in this study.** Lengths reported in bold are imputed using methods outlined in the text. Anatomical abbreviations: mc, metacarpal; mt, metatarsal. Taxonomical abbreviations: A, Ankylosauria; An, Ankylosauridae; C, Ceratopsidae; Ce, Centrosaurinae; *Cen*, *Centrosaurus*; Ch, Chasmosaurinae; *Cha*, *Chasmosaurus*; *Cor*, *Corythosaurus*; *Euo*, *Euoplocephalus*; *Gry*, *Gryposaurus*; H, Hadrosauridae; Ha, Hadrosaurinae; L, Leptoceratopsidae; La, Lambeosaurinae; *Lam*, *Lambeosaurus*; No, Nodosauridae; Oro, cf. *Ordodromeus*; P, Pachycephalosauridae; pac, ‘pachyrhinosaur’; *Pan*, *Panoplosaurus*; *Par*, *Parasaurolophus*; *Pro*, *Prosaurolophus*; Ste, *Stegoceras*; *Sty*, *Styracosaurus*; Une, *Unescoceratops*; *Vag*, *Vagaceratops*. Other abbreviations: MAZ, Megaherbivore Assemblage Zone; mc, metacarpal; mt, metatarsal. Estimated values reported in bold.

| Specimen | Suborder/ family | Family/ subfamily | Genus | Humerus | Radius | Ulna | Mc III | Femur | Fibula | Tibia | Mt III |
| --- | --- | --- | --- | --- | --- | --- | --- | --- | --- | --- | --- |
| ROM 784 | A | An | *Dyo* | **424** | 245 | ? | **116** | 544 | ? | ? | 108 |
| AMNH 5337 | A | An | *Euo* | **439** | 275 | ? | **121** | ? | ? | ? | ? |
| AMNH 5403 | A | An | *Euo* | 450 | 305 | 390 | 125 | ? | ? | ? | ? |
| AMNH 5404 | A | An | *Euo* | 420 | 271 | ? | **120** | 548 | ? | 412 | ? |
| AMNH 5405 | A | An | *Euo* | 447 | 260 | ? | **118** | ? | ? | ? | ? |
| AMNH 5406 | A | An | *Euo* | **423** | 243 | 312 | 116 | ? | ? | ? | ? |
| ROM 1930 | A | An | *Euo* | 415 | 215 | 350 | **112** | ? | ? | ? | ? |
| AMNH 5381 | A | No | *Pan* | 570 | **347** | ? | **128** | ? | ? | ? | ? |
| AMNH 5665 | A | No | *Pan* | 582 | 355 | 500 | 130 | ? | ? | ? | ? |
| CMN 2759 | A | No | *Pan* | 430 | **252** | ? | 110 | ? | 310 | 385 | ? |
| ROM 1215 | A | No | *Pan* | 420 | 245 | 350 | **109** | ? | ? | ? | ? |
| AMNH 5351 | C | Ce | *Cen* | 600 | 345 | 435 | 127 | 797 | 560 | 588 | 207 |
| AMNH 5427 | C | Ce | *Cen* | **735** | **445** | ? | 143 | 800 | 460 | 500 | 230 |
| ROM 1426 | C | Ce | *Cen* | 535 | 305 | 412 | 125 | 867 | 495 | 555 | 193 |
| ROM 767 | C | Ce | *Cen* | 538 | 343 | 418 | 118 | ? | ? | ? | ? |
| YPM 2015 | C | Ce | *Cen* | 592 | 297 | 439 | 120 | 788 | 519 | 545 | 190 |
| TMP 2002.076.0001 | C | Ce | pac | 682 | 395 | 530 | 159 | 600 | 483 | 510 | 136 |
| AMNH 5372 | C | Ce | *Sty* | 618 | 375 | ? | 140 | ? | ? | ? | 244 |
| CMN 344 | C | Ce | *Sty* | 575 | 378 | 483 | **130** | 818 | 577 | 626 | ? |
| CMN 2245 | C | Ch | *Cha* | 508 | 318 | 432 | 235 | 749 | 483 | 533 | ? |
| ROM 839 | C | Ch | *Cha* | 555 | 345 | 425 | 130 | 829 | ? | 547 | 202 |
| ROM 843 | C | Ch | *Cha* | 620 | 395 | 470 | 160 | 905 | 515 | 560 | 205 |
| CMN 41357 | C | Ch | *Vag* | 600 | 526 | 409 | 136 | 767 | 489 | 555 | 150 |
| MCSNM 345 | H | Ha | *Gry* | 730 | 680 | 785 | **211** | 1150 | ? | 1080 | 330 |
| TMP 1980.022.0001 | H | Ha | *Gry* | 540 | 502 | 520 | 224 | 955 | 815 | 860 | 316 |
| AMNH 5350 | H | Ha | *Gry* | ? | ? | ? | ? | 1140 | 930 | 1030 | **343** |
| ROM 764 | H | Ha | *Gry* | 600 | 560 | 615 | 220 | 1050 | 890 | 865 | 345 |
| ACM 578 | H | Ha | *Pro* | ? | ? | ? | ? | ? | ? | ? | ? |
| ROM 787 | H | Ha | *Pro* | 535 | 475 | 545 | 230 | 975 | 835 | 850 | 320 |
| TMP 1984.001.0001 | H | Ha | *Pro* | 560 | 540 | 584 | 261 | 1045 | 900 | 895 | **343** |
| AMNH 5240 | H | La | *Cor* | ? | ? | ? | ? | 1080 | 950 | 1000 | 380 |
| AMNH 5338 | H | La | *Cor* | 546 | 577 | 609 | 242 | 987 | 882 | 924 | 378 |
| CMN 8676 | H | La | *Cor* | 450 | 535 | 570 | 215 | 890 | 775 | 813 | 320 |
| ROM 845 | H | La | *Cor* | 530 | 640 | 695 | 270 | 1055 | 915 | 925 | 390 |
| TMP 1980.023.0004 | H | La | *Cor* | 546 | 616 | 671 | ? | 1145 | ? | 996 | 410 |
| TMP 1984.121.0001 | H | La | *Cor* | 530 | 640 | 650 | 255 | 1040 | ? | 980 | **349** |
| TMP 1980.040.0001 | H | La | *Cor* | 570 | 629 | 645 | 254 | 1070 | 905 | 949 | 394 |
| CMN 8703 | H | La | *Lam* | 520 | 616 | 660 | 265 | 1020 | 940 | 1000 | **336** |
| ROM 1218 | H | La | *Lam* | 500 | 600 | 665 | 250 | 1070 | 900 | 925 | 385 |
| TMP 1966.004.001 | H | La | *Lam* | 505 | 630 | 680 | 275 | 1063 | 976 | 1020 | 365 |
| TMP 1982.038.0001 | H | La | *Lam* | ? | 651 | 685 | 213 | 1125 | 1012 | 1067 | 420 |
| ROM 768 | H | La | *Par* | 495 | 490 | 540 | 185 | 1035 | ? | **909** | **424** |
| UALVP 300 | H | La | *Par* | 511 | ? | ? | ? | 991 | 885 | 870 | 406 |
| TMP 1995.012.0006 | L | ? | Une | **255** | **137** | ? | **45** | **265** | ? | **280** | **130** |
| UALVP 2 | P | ? | Ste | ? | ? | ? | ? | **235** | **220** | **225** | **105** |
| ? | ? | ? | Oro | ? | ? | ? | ? | **270** | **305** | **322** | **151** |
| **Specimen** | **Suborder/ family** | **Family/ subfamily** | **Genus** | **Tail** | **Trunk** | **Neck** | **Tail-Head** | **θ** | **Quadrupedal height** | **Bipedal height** | **MAZ** |
| ROM 784 | A | An | *Dyo* | ? | ? | ? | ? | ? | 786 | ? | 1 |
| AMNH 5337 | A | An | *Euo* | ? | ? | ? | ? | ? | 835 | ? | ? |
| AMNH 5403 | A | An | *Euo* | ? | ? | ? | ? | ? | 880 | ? | ? |
| AMNH 5404 | A | An | *Euo* | ? | ? | ? | ? | ? | 811 | ? | 1 |
| AMNH 5405 | A | An | *Euo* | ? | ? | ? | ? | ? | 825 | ? | ? |
| AMNH 5406 | A | An | *Euo* | ? | ? | ? | ? | ? | 782 | ? | ? |
| ROM 1930 | A | An | *Euo* | ? | ? | ? | ? | ? | 742 | ? | ? |
| AMNH 5381 | A | No | *Pan* | ? | ? | ? | ? | ? | 1045 | ? | 1 |
| AMNH 5665 | A | No | *Pan* | ? | ? | ? | ? | ? | 1067 | ? | 1 |
| CMN 2759 | A | No | *Pan* | ? | ? | ? | ? | ? | 792 | ? | 1 |
| ROM 1215 | A | No | *Pan* | ? | ? | ? | ? | ? | 774 | ? | 1 |
| AMNH 5351 | C | Ce | *Cen* | ? | ? | ? | ? | ? | 1072 | ? | 1 |
| AMNH 5427 | C | Ce | *Cen* | ? | ? | ? | ? | ? | 1322 | ? | ? |
| ROM 1426 | C | Ce | *Cen* | ? | ? | ? | ? | ? | 965 | ? | 1 |
| ROM 767 | C | Ce | *Cen* | ? | ? | ? | ? | ? | 999 | ? | 1 |
| YPM 2015 | C | Ce | *Cen* | ? | ? | ? | ? | ? | 1009 | ? | 1 |
| TMP 2002.076.0001 | C | Ce | pac | ? | ? | ? | ? | ? | 1236 | ? | 2 |
| AMNH 5372 | C | Ce | *Sty* | ? | ? | ? | ? | ? | 1133 | ? | 2 |
| CMN 344 | C | Ce | *Sty* | ? | ? | ? | ? | ? | 1083 | ? | 2 |
| CMN 2245 | C | Ch | *Cha* | 1651 | ? | ? | ? | ? | 1061 | ? | 2 |
| ROM 839 | C | Ch | *Cha* | ? | ? | ? | ? | ? | 1030 | ? | 2 |
| ROM 843 | C | Ch | *Cha* | 2400 | 1640 | 550 | ? | ? | 1175 | ? | 2 |
| CMN 41357 | C | Ch | *Vag* | ? | ? | ? | ? | ? | 1262 | ? | ? |
| MCSM 345 | H | Ha | *Gry* | 3770 | 2070 | 1100 | 6940 | 43 | 2560 | 4713 | 1 |
| TMP 1980.022.0001 | H | Ha | *Gry* | **3131** | 1690 | 830 | 5651 | 43 | 2131 | 3846 | 1 |
| AMNH 5350 | H | Ha | *Gry* | **3737** | ? | ? | **6778** | 42 | 2513 | 4559 | ? |
| ROM 764 | H | Ha | *Gry* | **3442** | 1620 | 990 | 6052 | 41 | 2260 | 3974 | 1 |
| ACM 578 | H | Ha | *Pro* | ? | ? | ? | ? | ? | ? | 5486 | 2 |
| ROM 787 | H | Ha | *Pro* | 3700 | 1720 | 875 | 6295 | 35 | 2145 | 3649 | 2 |
| TMP 1984.001.0001 | H | Ha | *Pro* | 4255 | 1900 | 2750 | 8905 | 32 | 2283 | 4778 | 2 |
| AMNH 5240 | H | La | *Cor* | 4130 | 1783 | 1173 | 7086 | 37 | 2460 | 4221 | 1 |
| AMNH 5338 | H | La | *Cor* | 3955 | 1505 | 1130 | 6590 | 35 | 2289 | 3814 | 1 |
| CMN 8676 | H | La | *Cor* | **3588** | ? | ? | **5725** | 34 | 2023 | 3227 | 1 |
| ROM 845 | H | La | *Cor* | 4300 | 1910 | 1210 | 7420 | 33 | 2370 | 4090 | 1 |
| TMP 1980.023.0004 | H | La | *Cor* | **4502** | 1720 | 1050 | 7272 | ? | 2551 | 4120 | 1 |
| TMP 1984.121.0001 | H | La | *Cor* | **4126** | ? | ? | **6944** | 35 | 2369 | 3987 | ? |
| TMP 80.40.1 | H | La | *Cor* | **4233** | 1895 | 1150 | 7278 | 35 | 2413 | 4149 | 1 |
| CMN 8703 | H | La | *Lam* | **4684** | ? | ? | **7306** | 30 | 2356 | 3675 | 1 |
| ROM 1218 | H | La | *Lam* | 4120 | 1810 | 1040 | 6970 | 35 | 2380 | 4026 | 2 |
| TMP 1966.004.001 | H | La | *Lam* | **4199** | 1600 | 1000 | 6799 | 36 | 2448 | 3964 | ? |
| TMP 1982.038.0001 | H | La | *Lam* | 3500 | 1920 | 1180 | 6600 | 48 | 2612 | 4925 | 1 |
| ROM 768 | H | La | *Par* | **4616** | 2030 | 1120 | 7766 | 31 | 2368 | 3983 | 1 |
| UALVP 300 | H | La | *Par* | 4420 | **1944** | **1072** | 7436 | 31 | 2267 | 3814 | 1 |
| TMP 1995.012.0006 | L | ? | Une | **870** | ? | ? | **1660** | **51** | **437** | **1288** | 2 |
| UALVP 2 | P | ? | Ste | 940 | 450 | 250 | 1640 | **37** | **?** | 986 | 1 |
| ? | ? | ? | Oro | **1480** | **578** | ? | 2300 | **30** | ? | 1155 | ? |

Data for the following specimens were taken from the sources cited below:

| **ACM 578: Anonymous (1937)** | **AMNH 5427: Brown (1917)** |
| --- | --- |
| AMNH 5240: Brown (1916) | CMN 8676: Sternberg (1935) |
| AMNH 5338 Lull and Wright (1942) | MCSNM 345: Pinna (1979) |
| AMNH 5350: Lull and Wright (1942) |  |
