# Supplementary material for: Feeding height stratification among the herbivorous dinosaurs from the Dinosaur Park Formation (upper Campanian) of Alberta, Canada
Source: BMC Ecol. 2013 Apr 4;13:14. doi: 10.1186/1472-6785-13-14 (PMC3637170; doi:10.1186/1472-6785-13-14)
Supplement: Additional file 2 — Reduced major axis regression results used to estimate missing elements in this study. [file 1472-6785-13-14-S2.doc]

Additional file 2

**Reduced major axis regression results used to estimate missing elements in this study**. Abbreviations as in Additional file 1.

| **Specimen** | **Suborder/ Family** | **Family/ Subfamily** | **Genus** | **Estimated element** | **Correlate** | **Equation** | **r^2^** |
| --- | --- | --- | --- | --- | --- | --- | --- |
| ROM 784 | A | An | Dyo | humerus | radius | y = 0.48599x + 305.31 | 0.50637 |
| ROM 784 | A | An | Dyo | mc III | radius | y = 0.1452x + 80.726 | 1 |
| AMNH 5337 | A | An | Euo | humerus | radius | y = 0.48599x + 305.31 | 0.50637 |
| AMNH 5337 | A | An | Euo | mc III | radius | y = 0.1452x + 80.726 | 1 |
| AMNH 5404 | A | An | Euo | mc III | radius | y = 0.1452x + 80.726 | 1 |
| AMNH 5405 | A | An | Euo | mc III | radius | y = 0.1452x + 80.726 | 1 |
| AMNH 5406 | A | An | Euo | humerus | radius | y = 0.48599x + 305.31 | 0.50637 |
| ROM 1930 | A | An | Euo | mc III | radius | y = 0.1452x + 80.726 | 1 |
| ROM 784 | A | An | Euo | humerus | radius | y = 0.48599x + 305.31 | 0.50637 |
| ROM 784 | A | An | Euo | mc III | radius | y = 0.1452x + 80.726 | 1 |
| AMNH 5381 | A | No | Pan | radius | humerus | y = 0.679x - 40.185 | 1 |
| AMNH 5381 | A | No | Pan | mc III | humerus | y = 0.1316x + 53.421 | 1 |
| CMN 2759 | A | No | Pan | radius | humerus | y = 0.679x - 40.185 | 1 |
| ROM 1215 | A | No | Pan | mc III | humerus | y = 0.1316x + 53.421 | 1 |
| AMNH 5427 | C | Ce | Cen | humerus | mc III | y = 8.215x - 440.08 | 0.098288 |
| AMNH 5428 | C | Ce | Cen | radius | mc III | y = 5.9606x - 407.67 | 0.008427 |
| CMN 344 | C | Ce | Sty | mc III | humerus | isometric |  |
| AMNH 5350 | H | Ha | Gry | mt III | femur | y = 0.14873x + 173.92 | 0.22057 |
| AMNH 5350 | H | Ha | Gry | tail | femur | isometric |  |
| AMNH 5350 | H | Ha | Gry | head-tail | femur | y = −1.386x + 8358.1 | 0.062608 |
| MCSNM 345 | H | Ha | Gry | mt III | femur | y = −0.0667x + 260 | 1 |
| ROM 764 | H | Ha | Gry | tail | femur | isometric |  |
| TMP 1980.022.0001 | H | Ha | Gry | tail | femur | isometric |  |
| TMP 1980.022.0001 | H | Ha | Gry | head-tail | femur | isometric |  |
| TMP 1984.001.0001 | H | Ha | Pro | mt III | femur | isometric |  |
| CMN 8676 | H | La | Cor | tail | femur | y = 3.5843x + 398.28 | 0.50743 |
| CMN 8676 | H | La | Cor | head-tail | femur | y = 5.5356x + 1186.9 | 0.50239 |
| TMP 1980.023.0004 | H | La | Cor | tail | femur | y = 3.5843x + 398.28 | 0.50743 |
| TMP 1984.121.0001 | H | La | Cor | mt III | femur | y = 0.35029x - 15.119 | 0.87766 |
| TMP 1984.121.0001 | H | La | Cor | tail | femur | y = 3.5843x + 398.28 | 0.50743 |
| TMP 1984.121.0001 | H | La | Cor | head-tail | femur | y = 5.5356x + 1186.9 | 0.50239 |
| TMP 1980.040.0001 | H | La | Cor | tail | femur | y = 3.5843x + 398.28 | 0.50743 |
| CMN 8703 | H | La | Lam | mt III | femur | y = 0.81985x-500.36 | 0.93213 |
| CMN 8703 | H | La | Lam | tail | femur | y = −11.273x + 16182 | 1 |
| CMN 8703 | H | La | Lam | head-tail | femur | y = −6.7273x + 14168 | 1 |
| TMP 1966.004.001 | H | La | Lam | tail | femur | y = −11.273x + 16182 | 1 |
| ROM 768 | H | La | Par | tibia | femur | isometric |  |
| ROM 768 | H | La | Par | mt III | femur | isometric |  |
| ROM 768 | H | La | Par | tail | femur | isometric |  |
| UALVP 300 | H | La | Par | neck | femur | isometric |  |
| UALVP 300 | H | La | Par | trunk | femur | isometric |  |
